# From Root to Seed: Unearthing the Potential of Carrot Processing and Comprehensive Utilization

**DOI:** 10.1002/fsn3.4542

**Published:** 2024-10-18

**Authors:** Haiyan Ding, Menglong Liu

**Affiliations:** ^1^ School of Public Health Dali University Dali Yunnan China

**Keywords:** by‐product, comprehensive utilization, *Daucus carota*, phytonutrient

## Abstract

Carrots, a globally cultivated root vegetable crop, are renowned for their nutritional and functional properties. However, the deep utilization and development of carrots and their derived products are limited in numerous countries, with a particular deficiency in advanced deep‐processing and transformation technologies. Consequently, the value of carrot products is diminished and resources are wasted. This review explores the nutritional value and characteristics of different carrot varieties, providing a comprehensive overview of the current state of carrot product processing. These products include primary processed products such as minimally processed carrots, dried carrots, pickles, preserves, fruit purees, yoghurt, juice, and essential oils, as well as processed by‐products including functional phytochemical extractions such as carotenoids, polyphenols, dietary fibers, active peptides, and glycoproteins. This review also covers other by‐products, including feeds, fast food boxes, and bioethanol production. Furthermore, cutting‐edge technologies related to carrot processing are discussed. The ultimate goal of this review is to provide researchers and practitioners with an in‐depth understanding of the present status of carrot production.

## Graphical abstract

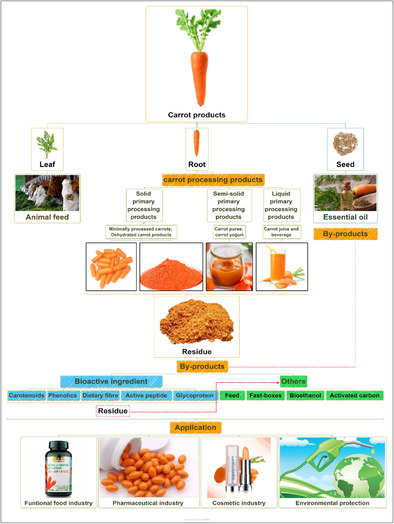

## 1 Introduction

Carrot (*Daucus carota* L.), a biennial flowering plant belonging to the family, is known for its crisp texture, fresh taste, and high nutritional value. Ranging as the world's second most popular vegetable, following potato (Que et al. 2019), carrots offer various nutrients and functional substances in the form of phytochemicals, such as dietary fiber, carotenoids, anthocyanins, vitamins, minerals, polyphenols, flavonoids, glycosides, alkaloids, saponins, and sesquiterpenoids, which are all vital for human health (Ismail et al. 2023). However, the global development and utilization of carrot products remain relatively limited. The lack of advanced technologies for deep processing and transformation results in diminished product value, waste of resources, and environmental pollution. To address these issues, it is crucial to strengthen the understanding of the development of the carrot industry, improve the utilization of carrot resources, and optimize processing techniques to maximize the retention of functional substances and achieve a sustainable resource cycle. This review offers insights into the current status and technological applications of carrot processing, aiming to promote the comprehensive utilization of carrot resources.

## 2 Carrot Classification and Nutritional Characteristics

The domesticated carrot is divided into two classifications: the Eastern/Asiatic group (var. *atrorubens*) and the Western group (var. *sativus*) (Swarup et al. 2023). Carrots grown in Eastern/Asian countries, known as anthocyanin carrots, are characterized by purple/black roots, a thicker and shorter conical shape, and pubescent branches and tend to flower early. In contrast, most Western carrots exhibit red, orange, and white roots, unbranched and sparsely hairy green leaves, regular biennial growth characteristics, and a low tendency to bolt without extended exposure to low temperatures (Que et al. 2019). Taxonomically, domesticated carrots have been segregated from wild carrots through several ranks: *D*. *Carota var*. *sativus* (*Hoffm*.), *D*. *Carota* subsp. *sativus* (*Hoffm*.) *Arcangeli*, and *D*. *sativus* (*Hoffm*.) *Roehl* (Nguyn 2015).

**FIGURE 1:**
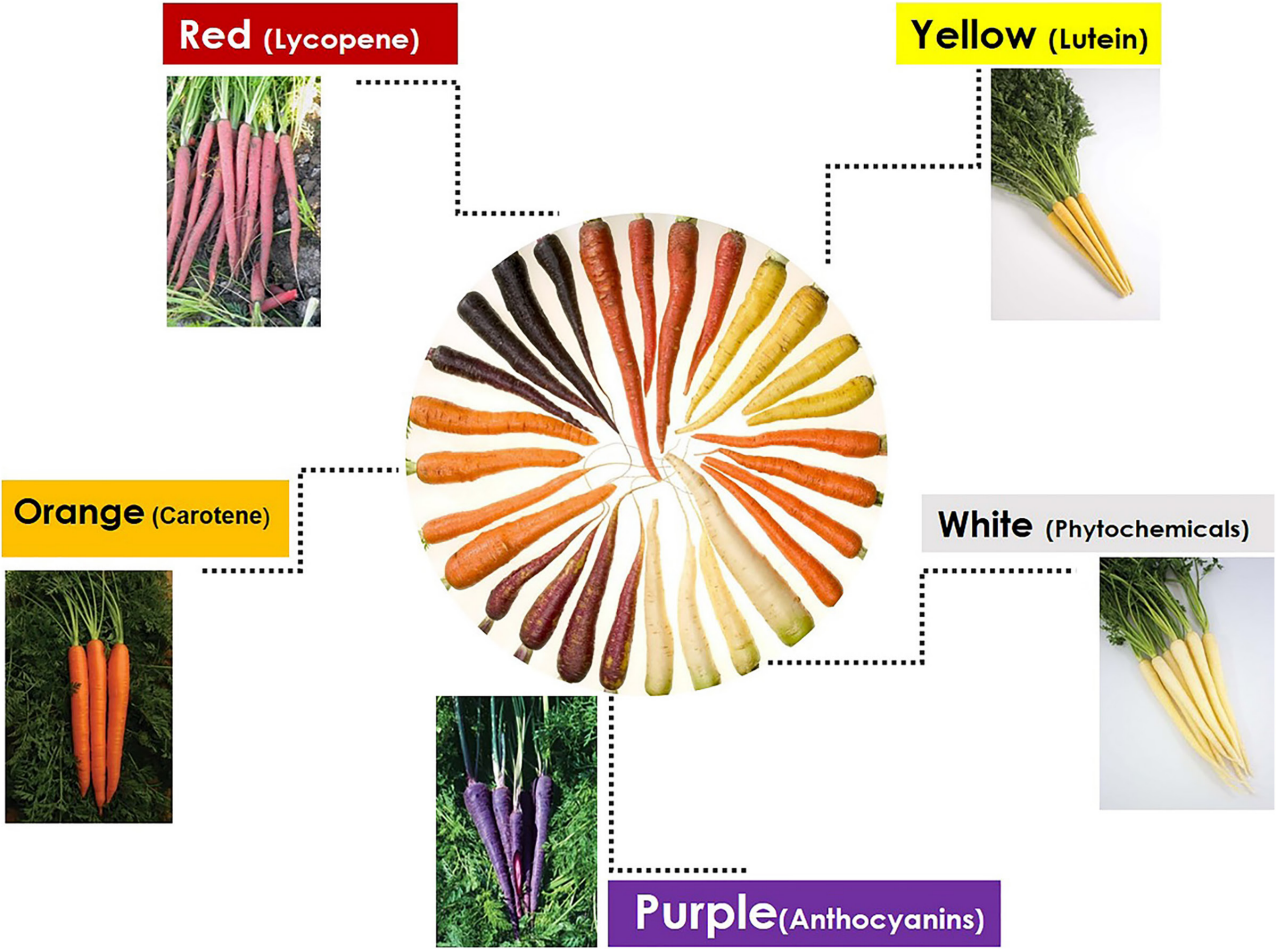
Phytonutrients rich in carrots of different colors.

Characteristics such as root color, shape, uniformity, texture, xylem phloem ratio, and internal bioactivity value of the fleshy edible roots are the primary selection criteria for its germplasm resources (Howarth et al. 2015; Kasale, Malagi, and Naik 2018). Wild carrots are typically white or light yellow in color, while domesticated cultivars are primarily orange or purple. Recently, new cultivars of carrots have been introduced to the global market in a range of colors, such as creamy‐white, light yellow, yellow, orange, orange‐red, red, violet, purple, and dark purple, and each color variation exhibits distinct variations in nutrition and flavor corresponding to its hue (Bhandari et al. 2022). The color difference in carrot varieties is based on the presence of pigments mainly carotenoids, lycopene, and anthocyanins (Varshney and Mishra 2022). White carrots contain very small amounts of pigments, yellow carrots contain lutein and some β‐carotene, orange and dark orange carrots are rich in lutein, α‐carotene, and β‐carotene, red carrots are rich in lycopene, while purple carrots are abundant in anthocyanins and phenolic compounds (Bhandari et al. 2022; Yusuf et al. 2021) (Figure 1). Carrot colors can be selected based on their specific processing and nutritional requirements for the production of carrot‐based products (Table 1). Orange carrots are the most prevalent and widely available variety used in manufacturing a wide range of products, including carrot juice, puree, soup, chips or crisps, powder, and baby food. Popular seed companies associated with orange carrots include Burpee, Baker Creek Heirloom Seeds, Johnny's Selected Seeds, Seeds of Change, Ferry‐Morse, Park Seed, High Mowing Organic Seeds, Seed Savers Exchange, Territorial Seed Company, Botanical Interests, Select Seeds, and West Coast Seeds. Purple carrots are also a good source of carotenoids, not only phenolic compounds, but can also be used to make purple carrot juice, puree, crisps, jams, jellies, and sauces. Well‐liked varieties such as Purple Haze, Cosmic Purple, Purple Dragon, and Amarillo Carrot are obtainable from companies such as Baker Creek Heirloom Seeds, High Mowing Organic Seeds, Burpee, and Johnny's Selected Seeds. Yellow carrots possess a mild flavor and are used to produce juice, puree, soups, dressings, marinades, and other infused products. Popular varieties such as Yellowstone, Yellowstone Hybrid, Amarillo, and Solar Yellow are available from seed suppliers such as Burpee, Baker Creek Heirloom Seeds, Johnny's Selected Seeds, Ferry‐Morse, Stokes Seeds, Select Seeds; Johnny's Selected Seeds, Botanical Interests, High Mowing Organic Seeds; Seed Savers Exchange, Territorial Seed Company, and Park Seed. White carrots are known for their sweeter and milder taste and can be used to process white carrot juice, puree, soups, and pickles. While white carrots are less common than other colored varieties, there are several popular options such as Lunar White from Baker Creek Heirloom Seeds, Adaptive Seeds, Seed Savers Exchange; White Satin from Johnny's Selected Seeds, High Mowing Organic Seeds and White Belgian from Territorial Seed Company and Botanical Interests. (Note: the above variety information comes from the official websites of various Internet companies).

**TABLE 1:** Nutritional components and functional active substances of different kinds of carrots.

Classification	Nutrition g/100 g DM	Active ingredients mg/100 g DM							Application	References
		Total dietary fiber mg/100 g DM	Total phenolics mg/100 g DM	Total carotenoids mg/100 g DM	Lutein μg/100 mg FW	α‐carotene μg/100 mg FW	β‐carotene μg/100 mg FW	Anthocyanin (mg cyanidin‐3‐O‐glucoside equivalent per 100 g of fresh weight)		
Purple	0 g fat, 31 g carbohydrates, 1 g fiber, 1 g protein	14–16	2314.2	19.07	176–224	ND	318–381	6372	Juice	Valerga et al. (2023); Yusuf et al. (2021)
Red	pomace: 4.86 g protein, 1.40 g fat, 65.8 g carbohydrates, 1030 mg Ca, 2340 mg K, 2100 mg Na	—	3846	22.32	—	—	—	6870	juice, puree, crisps, jams, jellies, and sauces	Megali et al. (2020); Yusuf et al. (2021)
Normal Orange	0.2 g total fat, 6.9 g carbohydrates, 2 g fiber, 0.7 g protein	14–19	435.9	14.36	60–180	1278–3131	3260–6653	563	Juice, puree, soup, chips, crisps, powder, baby food	Gajewski et al. (2007); Yusuf et al. (2021); Balkrishna et al. (2024)
Yellow	ND	13–22	100.4	36.14	138–232	14.43	332	ND	Juice, puree, soup, chips, crisps, powder, baby food	Gajewski et al. (2007); Yusuf et al. (2021)
White	ND	—	146.8	4.98	ND	2.88	ND	ND	juice, puree, soups, dressings, marinades	Yusuf et al. (2021)

*Note:* U.S. Department of agriculture: https://fdc.nal.usda.gov/fdc‐app.html#/food‐details/2258587/nutrients.

Abbreviations: DM, dry matter; FW, fresh weight; ND, not detected.

**FIGURE 2:**
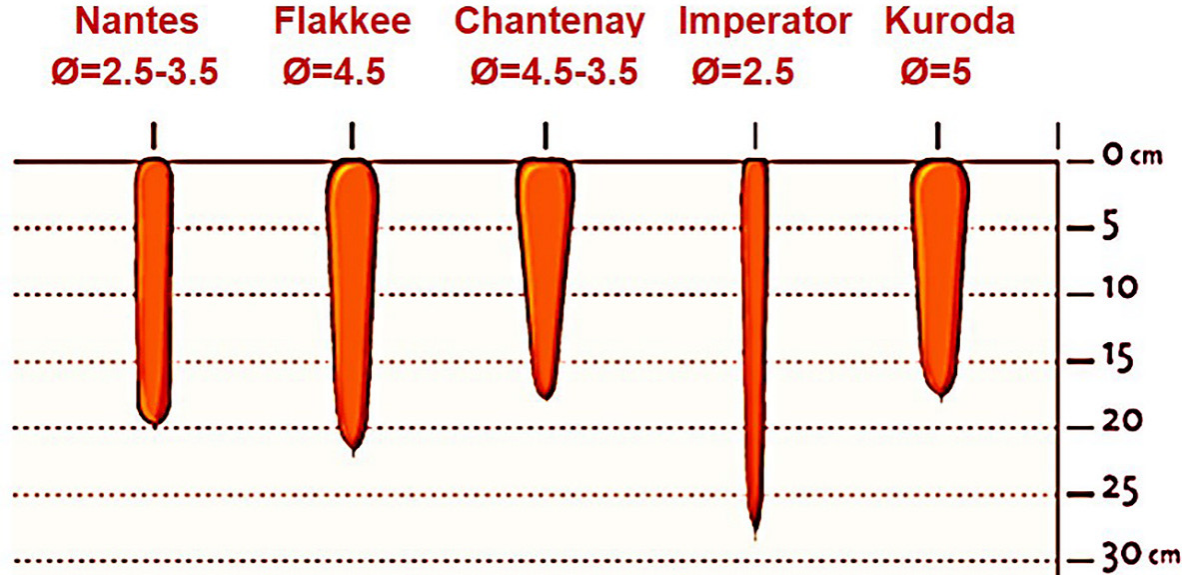
Carrot classification by root shape.

Carrots can be classified into five main categories based on the shape of their fleshy edible roots: Nantes, Flakkee, Chantenay, Imperator, and Kuroda (Figure 2). Carrot varieties exhibit distinguishing characteristics not only in their physical appearance but also in their taste, texture, and flavor. Nantes carrots have shorter and cylindrical roots that maintain a uniform shape from top to bottom, with blunt tips. Flakkee carrots possess a unique, cylindrical form with a broad shoulder and a tapered end, with a length ranging from 15 to 22 cm and a diameter ranging from 4–5 cm. In contrast, Chantenay carrots have a shorter and stockier structure with broader roots at the top that taper to a smaller, rounded tip. Imperator carrots exhibit a long, tapered shape that is wider at the top than at the tip. Kuroda carrots are typically cylindrical, with a length of approximately 15–20 cm and a diameter of 5–6 cm. Each type of carrot has distinct qualities that make it ideal for particular cooking and processing purposes. Carrot preferences differ among regions based on cultural and culinary practices. Asia had the largest planting area and greatest production from 2019 to 2021 (Table 2), and the Kuroda type is predominant in Asia (Wang et al. 2018), which is highly regarded for its sweetness and high yield and is commonly used in stir‐fries, salads, and pickling. Nates is the most common in Africa and Europe, and Imperator prevails in North America. Imperator carrots are widely used for the production of high‐value “cut and peel” or “baby carrots” due to their thinner and longer shape, accounting for nearly half of the US fresh market sales, and the convenience of this product has increased per capita carrot production slightly in the US (Simon 2019). The main producing area of Imperator in the United States is California, characterized by a dry climate and sandy loam soil, which are favorable for year‐round cultivation and processing of carrots (Zhuang, Hu, and Fang 2008). Additionally, the local fresh‐cut carrot industry has been developing for several years, with continuous optimization of products based on market feedback and ongoing updates of varieties to provide diversity. More than 10 companies are engaged in breeding Imperator carrots, namely, Nunhems, Vilmorin, Bejo, Seminis, Asgrow, Harris Morgan, Sakata, Sunseed, Prtoseed, Siegers, etc. (Zhuang, Hu, and Fang 2007), and the main varieties are UpperCut, CrispyCut, SugarSnax, HoneySnax, Highcut, Interceptor, Ibiza, etc. In addition to the United States, which has the largest cultivation area, imperator carrots are also grown in Canada and several European countries, including the Netherlands, France, and Germany. Furthermore, some companies grade Imperator carrots, which not only enhances the consistency of the final products but also makes the processed goods suitable for consumers of varying age groups.

**TABLE 2:** Annual data of carrot production in different regions from 2019 to 2021.

Area	Year	Area harvested (ha)	Yield (100 g/ha)	Production (t)	Import quantity (t)	Import value (1000USD)	Export quantity (t)	Export value (1000USD)
Asia	2019	623,985 (A)	409,556 (A)	25,555,693.67 (A)	805,726.14 (A)	342,040 (A)	1,198,012.24 (A)	510,825 (A)
	2020	608,722 (A)	419,402 (A)	25,529,922.31 (A)	835,222.91 (E)	356,205 (A)	1,167,213.97 (A)	465,139 (A)
	2021	624,593 (E)	422,198 (E)	26,370,168.37 (E)	931,160.32 (E)	486,228 (A)	1,116,554.73 (E)	547,799 (A)
Europe	2019	234,310 (A)	367,359 (A)	86,075,91.87 (A)	1,394,094.27 (A)	684,906 (A)	1,301,065.73 (A)	610,565 (A)
	2020	224,521 (A)	370,553 (A)	8,319,694.45 (A)	1,315,397.37 (A)	642,382 (A)	1,288,240.08 (A)	607,859 (A)
	2021	229,347 (A)	386,331 (A)	8,860,388.72 (A)	1325089.23 (A)	674,123 (A)	1,309,044.17 (A)	653,558 (A)
Africa	2019	120,625 (E)	188,584 (E)	2,274,801.13 (E)	65916.44 (A)	25,225 (A)	62,402.82 (A)	18,327 (A)
	2020	131,151 (E)	178,844 (E)	2,345,558.92 (E)	97939.57 (E)	33,497 (E)	80,248.18 (A)	20,653 (A)
	2021	121,615 (E)	193,874 (E)	2,357,798.3 (E)	132903.08 (E)	49,085 (E)	113,593.58 (A)	30,832 (A)
Americas	2019	112,320 (E)	333,096 (E)	3,741,316.71 (E)	398945.92 (A)	266,529 (A)	396,882.42 (A)	255,830 (A)
	2020	112,083 (E)	329,759 (E)	3,696,040.1 (E)	395575.04 (A)	259,402 (A)	396,294.41 (A)	247,096 (A)
	2021	113,349 (E)	325,634 (E)	3,691,014.74 (E)	414530.15 (A)	277,802 (A)	414,838.44 (A)	258,126 (A)
Oceania	2019	6758 (E)	578,712 (E)	391,103 (E)	6007.92 (E)	4181 (E)	107,041.13 (A)	64,824 (A)
	2020	6490 (E)	540,444 (E)	350,726.95 (E)	5,219.93 (E)	4004 (E)	109,891.55 (A)	68,567 (A)
	2021	7103 (E)	545,314 (E)	387,344.31 (E)	4,631.44 (E)	3891 (E)	103,617.83 (E)	68,954 (E)

*Note:* The above data from the website: https://www.fao.org/home/en/“E” and “A” in parentheses represent estimated values and official figures, respectively.

**FIGURE 3:**
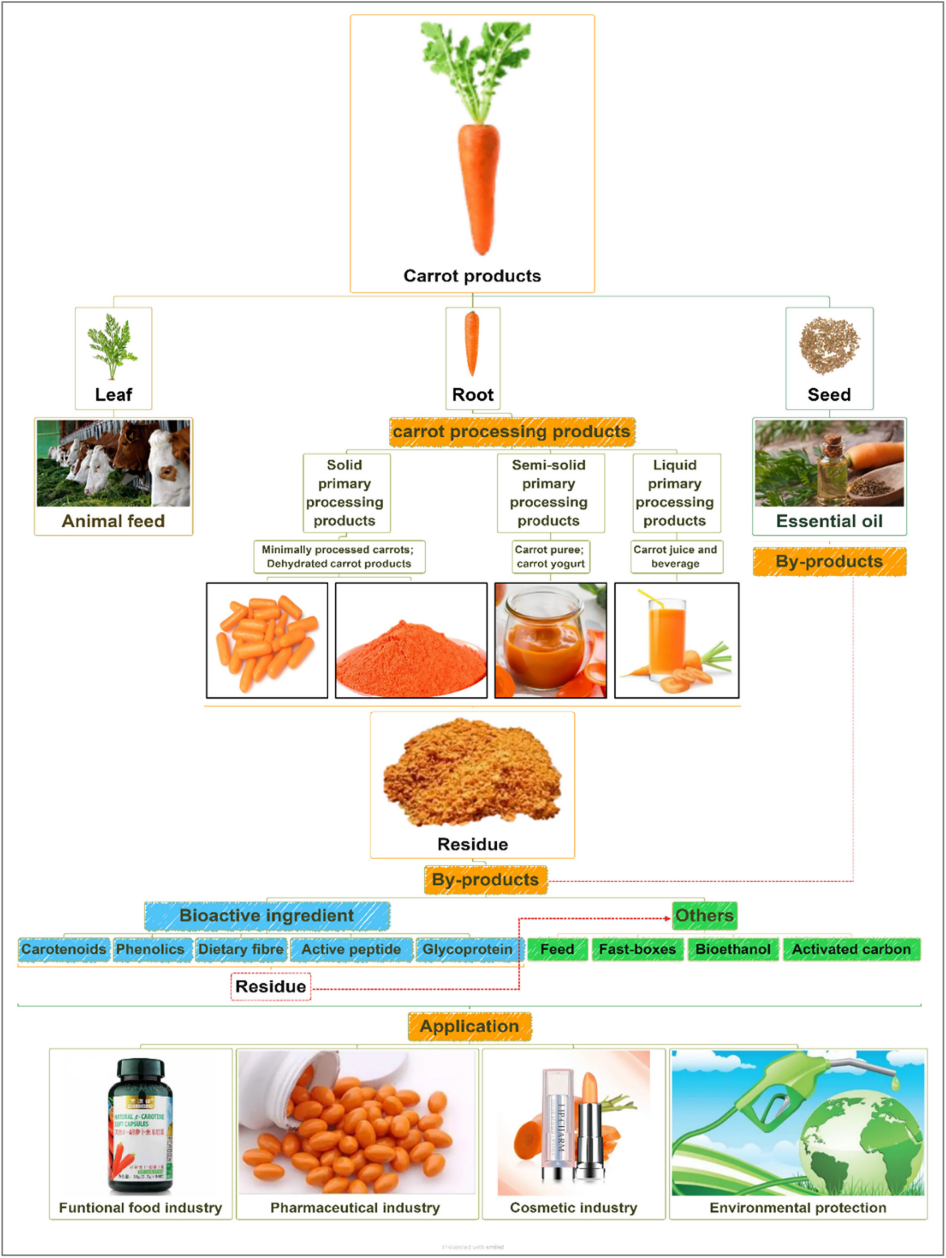
Carrot‐based products, by‐products, and their comprehensive utilization.

The following section will provide a comprehensive overview of the current situation of carrot product processing, covering both processed carrot products and by‐products. The primary processed products include minimally processed carrots and dried carrots, pickled carrots, candied carrots, carrot purees, yoghurt, juices, and essential oils. The by‐products involve the extraction and utilization of active ingredients, including functional chemical extracts such as carotenoids, polyphenols, dietary fiber, active peptides, and glycoproteins. Additionally, other by‐products include animal feed, fast‐food boxes, and bioethanol production (Figure 3).

## 3 Carrot Processing Products

### 3.1 Minimally Processed Carrots

With the accelerated pace of people's lives and the pursuit of high‐quality and healthy food, minimally processed vegetables and fruits have become increasingly popular with consumers due to their convenience, freshness, nutrition, and high edibility. To meet consumer demand, a new category of fresh carrots called minimally processed (MP) products has emerged, becoming widespread worldwide (Finger et al. 2023). Using fresh carrot roots as raw materials, these MP products undergo a series of processing steps, including sorting, washing, abrasion, peeling, cutting, grading, weighing, and packaging, to provide customers with fresh‐like and ready‐to‐eat products (Gastélum Estrada et al. 2020). The range of MP products includes carrot slices, dice, shreds, chunks, segments, balls, and other various shapes (Condurso et al. 2020; Piscopo et al. 2019; Ravula et al. 2017). Slices, dice, shreds, and chunks are mainly utilized as raw materials in the catering industry and are supplied to restaurants, canteens, and schools. In recent years, a new kind of MP product known as “baby carrot” in the USA market has been introduced and gained popularity among children and young people in Canada, some European countries, and some Asian countries, such as China and Japan. Preserving the quality of MP carrot products during storage and extending their shelf life pose significant challenges despite the relatively simple processing process. During storage, the nutritional and sensory qualities of MP carrots decrease due to factors such as water loss or microorganisms, which may affect consumer acceptance (Condurso et al. 2020). To mitigate microbial and chemical spoilage and extend the shelf life of MP products, various techniques are currently employed. These techniques include physical methods such as ultraviolet disinfection, ozone disinfection, ultrasonic cleaning, ultrahigh pressure, high‐pressure carbon dioxide treatment (Yu et al. 2019), modified atmosphere packaging (Ratajczak et al. 2022), temperature control, and irradiation technologies. Additionally, various chemical methods including chlorine washing (Caruso and Parisi 2016), hydrogen peroxide disinfection (Gastélum Estrada et al. 2020), acidic solution immersion disinfection (Piscopo et al. 2019), chemical coating (Wang et al. 2015) and antioxidant treatments are commonly employed in the production of MP products. However, post‐fresh‐cut washing or sanitizing procedures are potentially detrimental to the wound‐induced accumulation of health‐promoting compounds during storage (Valerga et al. 2023). As a recommended improvement strategy, it is suggested that fresh‐cut carrot products be disinfected only before cutting (Gastélum Estrada et al. 2020).

### 3.2 Dried Carrot Products

Drying is a traditional food preservation method worldwide since dried products can be used as a substitute for fresh fruits and vegetables in the off‐season. While thermal processing and the drying process indeed influence the distinct aroma of fresh carrots (Tian et al. 2024), the drying of carrots serves a multifaceted purpose. It not only safeguards them against microbial deterioration and enzymatic alterations, thereby extending their storage life and preservation period but it also contributes to a reduction in transportation costs to a certain degree (Nguyn 2015). At present, dried carrot products developed worldwide mainly include carrot slices (Singh and Gangwar 2020), carrot shreds and cubes (Chupawa et al. 2022; Sagar 2015), carrot granules, and carrot powder (Ariahu, Kamaldeen, and Yusufu 2021). Among these products, carrot powder is an important development direction in the processing of dried carrot products. The reason is that carrot powder has no strict requirements on the size and shape of the raw materials and can make full use of the nutrients and dietary fiber in the raw materials. Carrot powder not only includes pure carrot powder products but can also be added as a nutrient supplement to various foods, such as infant food (Roshana and Mahendran 2019), pasta (Porto Dalla Costa et al. 2016), bread (Pandey et al. 2024), cake (Salehi et al. 2015), dairy products, beverages and snacks (Ying et al. 2021), to make the products more nutritious and delicious. Edible carrot paper is also a new type of nutritional carrot product similar to the traditional seaweed cooking roll. It uses carrots as the base material and can also add other vegetables, such as potato and spinach powder (Qin et al. 2018; Ying et al. 2012). Subsequently, a blend of adhesives, including whey protein, collagen, CMC‐Na, and agar, among others, was incorporated, effectively binding the raw materials together. By employing strip‐forming techniques followed by hot air drying, edible carrot paper was crafted and imbued with specific mechanical resilience and nutritional attributes (Shang et al. 2012; Xiao and Li 2013).

How to retain carotenoids in dried carrot products to the greatest extent is a key issue in the production of these products (Ariahu, Kamaldeen, and Yusufu 2021). Carrot drying technology includes room temperature drying technology, solar drying techniques (Akter et al. 2024), hot air drying technology (Zhang et al. 2018), variable temperature and pressure differential puffing drying technology (Tabtiang, Umroong, and Soponronnarit 2022), ultrasound or microwave‐assisted drying technology (Ignaczak et al. 2023), and vacuum freeze drying (Feng et al. 2020; Ling et al. 2020; Lu et al. 2021). In terms of preserving the antioxidant activity of carrots to the greatest extent, microwave‐assisted drying technology is superior to hot air drying and vacuum freeze‐drying technology (Tabtiang, Umroong, and Soponronnarit 2022). In terms of retaining product flavor to the greatest extent, vacuum freeze‐drying technology is superior to other methods (Li, Wang, and Zhou 2023; Li et al. 2024). In addition to the importance of drying methods, appropriate pre‐treatment of drying materials as well as post‐drying treatment are vital components. During the process of dehydrating carrot products, the enzymatic degradation of bioactive compounds should not be underestimated. Lipoxygenases, which are the primary thermostable enzymes responsible for the degradation of carotene, can form free radicals that destroy carotenoids in carrots. Consequently, blanching raw carrot materials before drying can significantly reduce the activity of lipoxygenases and considerably enhance the color and overall sensory quality of dried products (Tabtiang, Umroong, and Soponronnarit 2022). Additionally, post‐drying treatments, such as coating, can also extend the shelf life of dried carrot products while ensuring that they remain intact (He et al. 2022).

### 3.3 Other Carrot Composition Processing Products

Other products, such as carrot preserves, carrot sausage, carrot cheese (Cankurt, Cavus, and Guner 2024), carrot peanut butter, carrot pickles, carrot noodles, and canned carrots, have also been introduced to the market, thereby enhancing people's daily lives (Singh, Srivastava, and Yadav 2021). Along with other vegetables, carrots can also be used to produce high‐quality pickles (Li et al. 2024). They can be preserved in non‐airtight containers using acidified brine with potassium metabisulfite at room temperature for up to 6 months. To preserve carrots, small whole carrots or carrot slices can be coated with sugar or heavy sugar syrup to increase the total soluble solids content to 70%–75%. The resulting product exhibits an appealing color, flavor, and texture. It can be stored at low temperatures (1°C–3°C) for 6 months while retaining 40% of its β‐carotene content (Sharma et al. 2012). Furthermore, carrot Halva stands as a beloved dessert in the northern reaches of India, where it enjoys immense popularity. Crafted meticulously with carrots as its cornerstone ingredient, it is artfully blended with milk, sugar, exotic spices, and oil to create a sensory delight. To cater to the convenience of Indian consumers and enhance their experience, the recipe for instant Halva undergoes continual refinement, ensuring it can be preserved seamlessly at −20°C, maintaining its freshness and flavor for longer periods (Kumar et al. 2020).

### 3.4 Carrot Puree and Other Semisolid Primary Processing Products

Due to consumer demand for diverse and high‐quality products, carrot roots can be processed into carrot puree, which is commonly used as a main ingredient in plant‐based foods (Özcan and Yıldız 2016). Gradually introducing different textures is essential in infant feeding practices. The smooth and readily digestible texture of carrot puree makes it an ideal introductory offering for infants embarking on their culinary journey. It can also be used as a healthy companion to various meals, enhancing their taste and color when combined with diverse ingredients such as vegetables, meats, or flour to make salads, soups, stews, or noodles (Prerana and Anupama 2020). Furthermore, carrot puree can serve as a supplementary source of nutrients (Gomaa, Gomaa, and El‐All 2021) in the diet, benefiting individuals with special dietary requirements by supporting immune function, improving digestive health, and aiding in the reduction of cholesterol levels. Additionally, carrot yoghurt is also a prevalent semisolid primary processing product made from milk and white sugar as primary ingredients, with various forms of carrot added, including carrot pulp, juice, waste (Šeregelj et al. 2021), pomace powder (Stoica et al. 2024), fiber powder (Dong et al. 2022; Vénica et al. 2020), extract (Turturică et al. 2021), and concentration (Pandey et al. 2021). This formulation represents a strategy for the design of functional dairy beverages with probiotics (Munteanu‐Ichim et al. 2024).

### 3.5 Carrot Juice and Beverages

Carrot juice and its derivatives, including pure or blended fruit and vegetable beverages, milk beverages containing carrot juice, and fermented carrot lactic acid bacteria beverages, rank among the most popular non‐alcoholic beverages (Kiros et al. 2016). The antioxidant activity of juice obtained from carrots of different colors varied. In particular, black carrot juice exhibited superior antioxidant activity compared to yellow and orange carrot juice (Purkiewicz et al. 2020). The extraction yield of carrot juice is influenced by numerous factors, such as carrot variety, degree of crushing of the raw carrot materials, juice extraction method, extraction process, filtration method, and disinfection method (Aliyev et al. 2021; Tanguler et al. 2021). Currently, there are various methods for obtaining carrot juice, mainly including mechanical pressing, high‐pressure processing, cold pressing, enzyme‐assisted extraction, solvent extraction, and supercritical carbon dioxide methods. These methods provide various options for obtaining carrot juice with different extraction rates, each offering distinct advantages and disadvantages related to yield, quality, and processing complexity. Mechanical pressing achieves an extraction rate of approximately 70%–80% with the advantages of preserving nutritional value, a simple process, and minimal heat generation. However, it has lower yields than other methods and has higher equipment costs (Catania et al. 2020; Wilczyński et al. 2020). The high‐pressure processing technique for carrot juice preserves nutrients, extends shelf life, and maintains color and flavor. Nonetheless, it entails high equipment costs, limited effectiveness against some pathogens, and longer processing times than traditional methods, which may affect production efficiency (Szczepańska et al. 2021). Enzyme‐assisted extraction results in an extraction rate of approximately 80%–85% and offers higher yield, improved nutrient retention (Liu et al. 2023), and reduced energy consumption. However, it involves additional processing steps and may have a potential impact on texture, color, and flavor. Solvent extraction boasts an extraction rate of approximately 90%–95% and provides high yield, rapid extraction, and suitability for the extraction of certain compounds. Nevertheless, it raises concerns about residual solvent, the potential impact on flavor and safety, and the need for additional purification steps (Miękus et al. 2019).

Additionally, to prolong the shelf life of carrot juice, effective sterilization technology is necessary. Among various sterilization technologies, ohmic sterilization technology has been found to retain a greater nutritional value of carrot juice than traditional heat sterilization technology (Debbarma et al. 2021; Negri Rodríguez et al. 2021). The combination of ultrasound and mild temperature can effectively decrease the microbial load in carrot juice to a safe level, making it beneficial for the industrial processing of carrot juice without changing the quality attributes or bioactive compounds (Lepaus et al. 2023). Additionally, non‐thermal emerging technologies, including ultrasound, high‐pressure processing, pulsed electric fields, ionizing radiation, and atmospheric cold plasma, have been extensively utilized as alternative methods to preserve food (Hernández‐Hernández, Moreno‐Vilet, and Villanueva‐Rodríguez 2019; Starek‐Wójcicka et al. 2023). Research has demonstrated that, when compared to heat treatment, non‐thermal treatment methods, particularly high‐pressure treatment technology, exhibit superior performance in maintaining product stability and quality retention (Bansal et al. 2019; Hwang et al. 2023). However, the selection of sterilization technology for carrot juice in practical production requires considering the composition, formulation, and physical state of the raw materials, and the behavior of various colloidal systems, as these factors significantly impact the efficiency of sterilization. Furthermore, the production cost is also a crucial criterion that affects selection. Even though processing technology can lead to the loss of some nutritional components in carrot juice, recent research has indicated that harnessing traumatic stress to boost the content of bioactive elements, such as phenolics, in fruits and vegetables offers a promising strategy for enhancing the health benefits of fruit and vegetable products. This presents an intriguing and beneficial direction for the production of carrot juice (Gastélum‐Estrada et al. 2023).

### 3.6 Essential Oil

A light yellow to reddish‐brown essential oil with a pleasant aroma is obtained when the crushed seeds of carrots are steam‐distilled (Attokaran 2017). Carrot essential oil has been used as a natural food colorant or blended with other plant oils to produce high‐value products. In addition, it is also utilized in the pharmaceutical, nutraceutical, or cosmetic industries for various applications, such as skin protection from certain diseases, attributed to its biological activities, such as antimicrobial, antibacterial, antifungal, herbicidal, antioxidant, and anticancer properties (Singh et al. 2023). Carrot essential oil is generated and concentrated within specialized secretory ducts commonly known as vittae. This versatile oil can be sourced from multiple facets of the carrot plant (Stanojević et al. 2023), encompassing its roots, leaves, and seeds. Notably, the seeds of the carrot plant are particularly abundant in this oil, boasting concentrations ranging from 0.8% to 1.6% (v/w), as meticulously recorded by Chizzola (2010). Carotol stands as the preeminent constituent in both carrot seed edible oil, comprising 30.55%, and seed essential oil, where it dominates at 66.78%, according to the latest findings by Paparella et al. (2024). In addition to carotol, the essential oil extracted from carrot juice is also abundant in sabinene (12.80%), β‐caryophyllene (8.04%), and α‐pinene (6.05%), making it a highly sought‐after ingredient in both pharmaceutical and food industries (Ma et al. 2015). Meanwhile, the essential oils isolated from the flowers, leaves, and stems of the carrot plant showcase a distinct chemical profile, dominated by asarone (ranging from 9.8% to 9.4%), α‐pinene (10.9% to 10.6%), and β‐bisabolene (7.6% to 9.3%), as elegantly illuminated by Mohammedi et al. (2015). This intricate blend of compounds underscores the rich biochemical diversity within the carrot plant and highlights its potential for various applications and industries.

## 4 Active Ingredients in Carrot Pomace and Other By‐Products

Carrot is an ideal raw material for cooking and processing, however, approximately 25% of the annual production is regarded as by‐products due to strict market policies (Kee et al. 2021). Following primary or deep processing, carrot reverts material and pomace retain significant levels of valuable edible substances and bioactive phytochemicals, such as dietary fiber, carotenoids, anthocyanin (Hernández‐Acosta et al. 2024), polyphenols, eugenin, luteolin, etc., demonstrating this waste's variety and prospective applications (Ikram et al. 2024; Nahar et al. 2024; Almatroodi et al. 2024). Landfill disposal of these wastes leads to significant environmental issues due to the emission of greenhouse gases such as methane and carbon dioxide (Kaur, Subramanian, and Singh 2022). Carrot waste and by‐products possess the potential to undergo additional processing, resulting in the production of edible foods or value‐added products that offer notable functional and nutritional benefits. Consequently, further development and exploitation of the nutritional and functional components in carrot residue, which can improve its economic value and reduce environmental pollution are recommended (Bas‐Bellver et al. 2023).

### 4.1 Carotenoids

Carotenoids are a group of phytochemicals predominantly synthesized in plants, cyanobacteria, algae and certain bacteria and fungi, comprising more than 700 carotenoids in nature (González‐Peña et al. 2023). However, only approximately 40 carotenoids are present in the human diet, of which α‐carotene, β‐carotene, lycopene, lutein, and β‐cryptoxanthin make up almost 90%, and the carotenoid content is ranked as yellow carrot > purple root > orange root > white root (Yusuf et al. 2021). Carotenoids can be categorized into two groups: (1) those with pro‐vitamin A activity, including β‐carotene, α‐carotene, and β‐cryptoxanthin, and (2) those without pro‐vitamin A activity, including lycopene, lutein, and zeaxanthin (Bhatt and Patel 2020). The abundant presence of antioxidant carotenoids in carrots is thought to contribute to their biological and medicinal properties. The regular consumption of carotenoids has been shown to have protective effects against certain types of cancer (Chen et al. 2021), diabetes associated with obesity and hypertension cataract formation (Marcelino et al. 2020), age‐related macular degeneration (Johra et al. 2020) cardiovascular disease and Alzheimer's disease (Bhatt and Patel 2020; González‐Peña et al. 2023). Carotenoids, particularly carotene, also possess effective coloring properties due to their thermal stability and are widely used in the production of various foods, such as high‐fat foods, candy, ice cream, and fruit processing (Luzardo‐Ocampo et al. 2021). Additionally, carotenoids extracted from carrot waste, when incorporated into a biopolymer matrix, serve as an important component in the development and utilization of “intelligent biodegradable film” for monitoring the spoilage and quality of meat products, seafood, milk, and other food items (Bhargava et al. 2020). To extract carotenoids from carrots, orange, and deep orange carrots are preferred because they have relatively high carotenoid contents. The total carotenoid content in the edible part of carrot roots ranges between 3.2 and 170 mg/kg (Ahmad et al. 2019). Commercial carotenoids are commonly synthesized using chemical methods or extracted from plant sources using petrochemical solvents. However, the traditional approach of extracting carotenoids through petrochemical solvents has several limitations, including long extraction periods, high temperatures, harmful volatile organic compound emissions, low extraction yields, and high residual waste. In recent years, alternative extraction techniques have been explored to address these limitations. These techniques include supercritical fluid extraction (SCFE), subcritical water extraction (SCWE), ultrasonic‐assisted extraction (UAE), microwave‐assisted extraction (MAE), pulsed electric field (PEF), and high hydrostatic pressure (HHP) (Kaur, Subramanian, and Singh 2022; Rodríguez, Sheila, and Vijaya 2021). Each technique has advantages and limitations and is influenced by many factors, such as temperature, pH, and electromagnetic wave frequency. Table 3 demonstrates the advantages and limitations of these techniques. When selecting the most suitable technology for the extraction of carrot bioactive compounds, various factors should be considered according to the nature of the extracted components, the processing conditions, and other factors. Supercritical fluid extraction (SCFE) using CO_2_ and ethanol as solvents is a particularly appropriate method for extracting carotenoids from carrots, with a potential extraction rate of up to 90% (De Andrade et al. 2019). Compared with conventional solvent extraction (CSE) and ultrasound‐assisted extraction (UAE), the microwave‐assisted extraction (MAE) method is also highly effective at extracting carotenoids and polyphenols from carrots, yielding a total extraction amount of 192.81 mg/100 g DW of carotenoids and 78.12 g GAE/100 g DW of total phenols (Kaur, Subramanian, and Singh 2022). The synergistic combination of multiple techniques not only enhances efficiency but also contributes to cost‐effectiveness and reduced environmental impact. For instance, through the concurrent extraction of supercritical CO_2_ and organic solvents, the extraction efficiency of carotenoids can be significantly improved from 86.10% to 96.20% (De Andrade Lima et al. 2018).

**TABLE 3:** Pros and cons of techniques for extracting natural compounds from fruits and vegetables.

Methods	Pros	Cons	References
Microwave‐assisted extraction (MAE)	Easy operation, high efficiency, time saving, solvent saving; Suitable for the extraction of temperature sensitive and volatile compounds. Samples can be heated quickly and uniformly	It may cause problems such as thermal decomposition of the sample, uneven heating of the sample, and high equipment; Under some operating conditions, the loss rate of the components may be increased	Ethaib et al. (2020); Ren et al. (2022)
Ultrasonic‐assisted extraction (UAE)	High extraction efficiency, can reduce the amount of solvent; The extraction effect is stable and easy to control; The extraction is fast and easy to operate	The extraction time is long, and the process is not easy to master. There are more residual substances after extraction	Naik et al. (2021)
Pulsed electric field (PEF)	High extraction efficiency; avoids some problems caused by the formation of ozone in the traditional heating method	Equipment is expensive; it may be limited by the safety problems of high voltage	Alam et al. (2018); López‐Gámez et al. (2020)
High hydrostatic pressure (HHP)	The extraction efficiency is high, and the activity and nutrients of biological molecules like enzymes and cells can be better retained. Low cost	Need longer processing time; High‐voltage equipment required	Jeż et al. (2019)
Supercritical fluid extraction (SFE)	It has the advantages of being nontoxic, no pollution, easy recovery, and high efficiency. It can be used for deep extraction of high molecular weight compounds	High technical requirements, the need to use the appropriate extraction agent and maintain the appropriate temperature parameters; The equipment cost is high	Aniceto et al. (2022); Silva et al. (2016); De Andrade Lima et al. (2018)

### 4.2 Phenolics

Carrots are a good source of phenolic compounds that are primarily distributed in the roots but are highly concentrated in the periderm tissue. The specific content of phenolic compounds in carrots of different colors will vary due to factors such as variety, planting conditions, and maturity. In general, purple‐black carrots have the most types and contents of phenolic substances, followed by orange, yellow, and white (Bhandari et al. 2022), and the phenolic content in purple carrot can reach 33.25 mg/g dry matter (Ma et al. 2020). The main phenolic compounds found in purple‐black carrots are chlorogenic acid, caffeic acid, ferulic acid, 4‐coumaric acid, and 4‐vinylphenol, with chlorogenic acid being the most abundant, accounting for 42.2%–61.8% of the total phenolic compounds. In terms of decreasing phenolic content, the order in different carrot tissues was epidermis > phloem > xylem. Although carrot peel constitutes only 11% of the fresh weight of carrots, it provides 54.1% of the total phenolic compounds, while the phloem tissue contributes 39.5%, and the xylem provides only 6.4%. The decrease in antioxidant and free radical scavenging activity in carrots followed the same pattern as the decrease in phenolic content (Sun, Simon, and Tanumihardjo 2009). Therefore, the increased phenols and antioxidant properties of carrot peels, which are regarded as waste in the processing industry, can be considered for value‐added utilization.

### 4.3 Dietary Fiber

Dietary fiber is an indigestible complex carbohydrate found in the structural components of plants. It cannot be absorbed by the body and therefore has no calorific value. A fiber‐rich diet is associated with severe health problems, a reduction in high cholesterol levels, the prevention of certain forms of cancer, and an impact on the gut microbial ecology and host physiology (Makki et al. 2018). The dietary fiber in carrots is composed of pectin, cellulose, hemicellulose, and lignin, with contents of 7.41%, 80.94%, 9.14%, and 2.48%, respectively (dry matter). Dietary fibers are desirable not only for their nutritional properties but also for their functional and technological properties, making them suitable as food ingredients. For instance, carrot residue can be incorporated into barley flour and processed using extrusion and steaming techniques to produce high‐fiber instant puffed snacks (Shirazi et al. 2020). Furthermore, the addition of carrot fiber to dairy products can enhance their nutritional value and richness (Say, Saydam, and Güzeler 2022). Additionally, adding it to sausages can increase the dietary fiber content and improve the sensory characteristics of the product (Alvarado‐Ramírez et al. 2018). Consequently, the addition of carrot dietary fiber can diversify the market for processed foods.

### 4.4 Active Peptide

Extracts from carrot seed and its essential oil have been reported in experimental studies to have cardio‐ and hepato‐protective effects, cognitive dysfunction, cholesterol‐lowering, antibacterial, anti‐fungal, anti‐inflammatory, analgesic, wound healing, and fertility benefits (Choe et al. 2018; Dias 2014; Priyanka and Khanam 2019; Sadeghi et al. 2020), these extracts could be applied in functional food and viewed as the main element of dietary supplements for human consumption to increase their value in the market. To date, there has been research on small chemical compounds and essential oils and few reports on proteins and their hydrolysates from carrot seeds (Śmigielski et al. 2014a, 2014b). The search for antioxidant peptides from natural sources and food has gained increasing interest among researchers. Enzymatic hydrolysis is mostly used in the preparation of antioxidant peptides in food industries to obtain active peptides from carrot seeds. The necessary steps include extracting total protein, hydrolyzing proteins, and purifying proteins. The key step is to obtain the optimum hydrolysis conditions, including hydrolysis time, substrate concentration, and protease dosage, and then the peptides with stronger activity need further purification by gel filtration chromatography in the last step (Ye et al. 2018).

### 4.5 Glycoproteins

Research has shown that residues from carrot extracts are rich in glycoproteins, which can be used in antioxidant and antiaging materials due to their strong biological activity. These glycoproteins eliminate reactive oxygen species, protect the cell membrane, and serve as antioxidants and antiaging agents in skin exposed to solar ultraviolet light (Lee, Jeong, and Jang 2015). Carrot glycoproteins are composed of 94.2% glycoproteins and 2.35% carbohydrates, and their amino acids contain small amounts of hydroxyl‐proline and glycine, which are characteristic of collagen peptides, and large amounts of glutamic acid and aspartic acid are involved in glucose and fat metabolism (Lee, Jang, and Jeong 2013).

### 4.6 Other By‐Products

Carrots and their by‐products can be used as promising alternative sources of green fodder, improving feed conversion efficiency by 25% (Forwood et al. 2023). Carrot straw, a high‐quality silage feed, is a natural source of antioxidants and nutritional supplements that are rich in carbohydrates, proteins, and minerals, including high crude protein (144 g/kg), calcium (24.3 g/kg) and phosphorus (7.7 g/kg) (Muwakhid, Kalsum, and Wati 2021). The use of carrot leaf extract in the diet can significantly improve the feed conversion rate of broilers during the fattening period (Utami et al. 2023). In addition, by mixing carrot residue with a thickener and undergoing drying and coating processes, it can be transformed into various by‐products, such as fast‐food boxes. Repurposing these fast‐food boxes as animal feed helps minimize environmental pollution and contributes to resource conservation. The production of fast boxes from carrot residue involves critical technologies such as the use of a thickening agent and plasticization processes. Moreover, carrot waste is a promising raw material for bioethanol production, with each ton producing 77.5 L of ethanol (Aimaretti et al. 2012), and the ethanol content of the distilled product is as high as 92.48% (v/v) (Palacios‐Velásquez et al. 2023). The key challenge in making bioethanol from carrot slag is to improve the polysaccharide hydrolysis of underutilized biomass, including optimizing glycation fermentation conditions and screening fermented microorganisms to improve bioethanol production (Demiray et al. 2016). Moreover, compared to high‐sugar feedstocks such as sugarcane or molasses, the ethanol industry has displayed limited interest in utilizing carrot waste for ethanol production. Therefore, it is necessary to study comprehensive recovery methods for the various bioactive substances present in carrot waste, including carotenoids, cellulose, and ethanol (Clementz et al. 2019). Additionally, activated carbon synthesized from carrot waste has potential as a cost‐effective organic adsorbent for treating water pollution (Moradi et al. 2021).

Currently, there are numerous primary processing products and functional components of carrots, ranging from seeds to edible roots, developed globally. However, when evaluated from the perspectives of market demand, processing status, product yield, and environmental protection, the most common types of carrot processing products are still fresh food, carrot juice, dried carrots, and other products. These product types not only meet the needs of the market and consumers, but also have more mature production conditions, processing technology, and logistics transportation. However, with consumers' increasing attention to product diversification and nutritional needs on the market, how to improve the processing yield of these primary processed products, retain the active ingredients in carrot raw materials to the greatest extent, reduce the use of additives in processed products, understand the influence of processing technology on the active ingredients of the product, and address biological pollution and other technological aspects still need continuous attention.

## 5 Research Status of Carrot and Future Perspectives

To provide a comprehensive overview of the current research landscape in the carrot industry, we conducted an exhaustive literature search on the Web of Science website (https://webofscience.clarivate.cn/wos/alldb/basic‐search), employing “carrot” as the search query (topic) and spanning the past five years from January 2022 to August 2024. This search yielded a total of 4052 pertinent articles, indicative of the significant research output within this field. The research fields encompassed by these carrot‐centric articles exhibit a marked concentration in agriculture (2031), plant sciences (1966), food science and technology (1891), nutrition and dietetics (1236), environmental sciences and ecology (1227), chemistry (1204), biochemistry and molecular biology (1120), science and technology—miscellaneous (999), engineering (775), and genetics and heredity (757). This distribution underscores the global academic community's primary research interest in carrots within the agricultural and food sectors.

**FIGURE 4:**
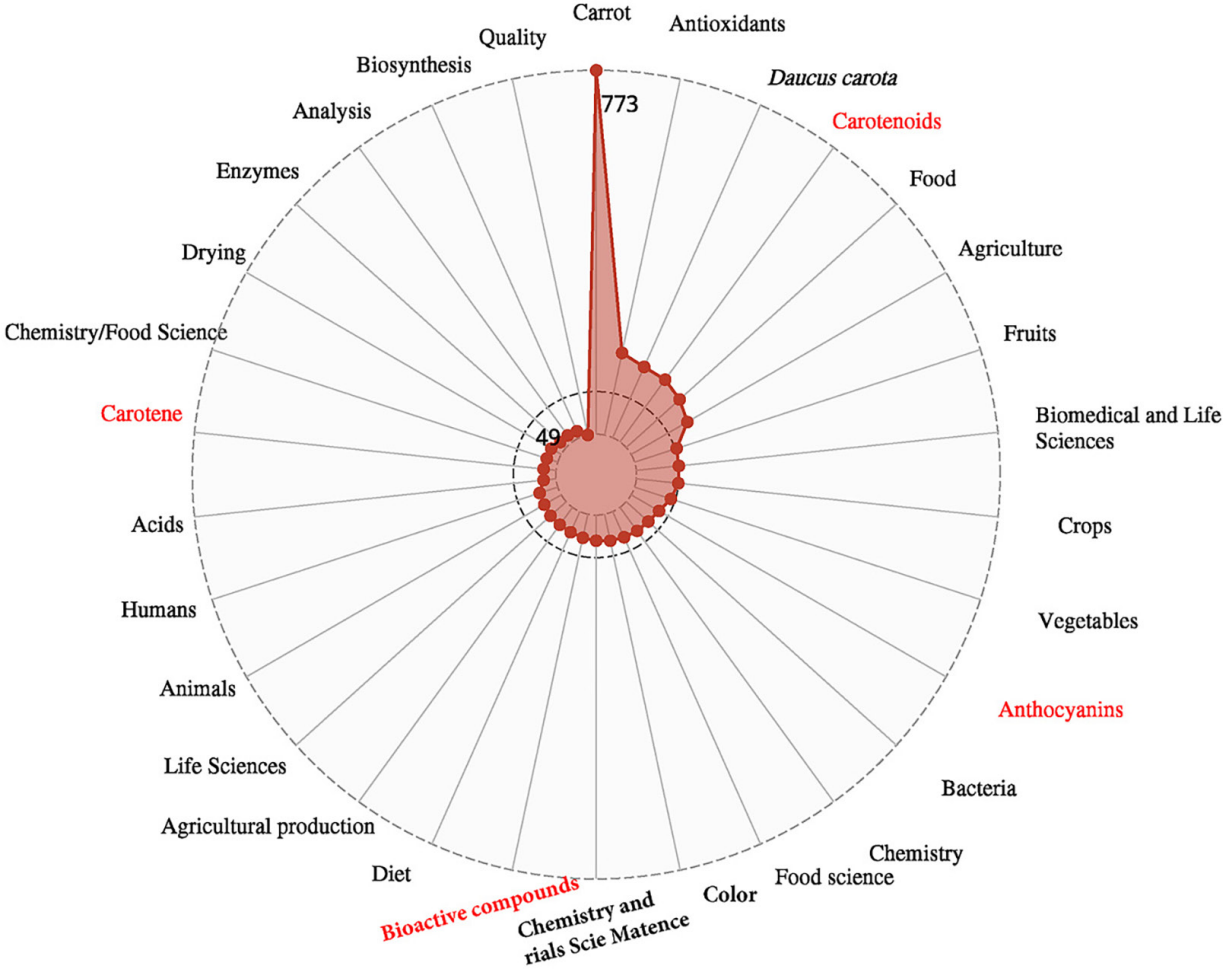
Hot words related to carrot study in the past 5 years from 2019 to 2023.

To delve deeper into the specifics within these broad categories, we exported the bibliographic details of these articles into the NoteExpress literature management software (version 4.0, Beijing Aiqin Haile Technology Co., Ltd.). Subsequently, we performed a rigorous statistical analysis of the keywords extracted from these 4052 articles, using “keywords” as the metric. By selecting the top 30 most frequently occurring keywords, we conducted a frequency analysis and visualized the results in a radar plot. This visualization was achieved utilizing the R software (version 4.2.2), specifically the “ggradar” (version 0.2) and “ggplot2” (version 3.4.2) packages, facilitated by Hiplot Pro (https://hiplot.com.cn/), a versatile web‐based platform for biomedical data analysis and visualization (Figure 4). The top 30 keywords with the highest frequency range from 49 to 773, with “carrot” being the most prominent. Overall, the biomedical potential, food development opportunities, and diverse aspects of the agricultural domain related to carrots—including genetic breeding, pest and disease control, and root quality assurance—emerge as the core research themes for scholars. Notably, carrots have garnered substantial attention in the biomedical realm owing to their exceptional antioxidant properties. Researchers' focus is intently directed toward the bioactive components abundant in carrots, particularly anthocyanins, carotenoids, and carotenes. These components not only underscore the health benefits of carrots but also signal their vast potential for applications in medicine and healthcare.

However, numerous challenges still exist in both carrot cultivation and processing, as well as in the comprehensive utilization of carrots (Paparella et al. 2024). Specifically, inadequate carrot cultivars and a lack of mature, high‐yield, and high‐quality cultivation techniques hinder the processing of various carrot products. For instance, carrot varieties suitable for juicing should possess a vibrant color, appropriate sweetness, abundant nutrients (vitamins, minerals, and antioxidants), and a crisp, tender texture with low cellulose content, thereby ensuring a bright color, pleasing taste, and high juice yield in the final product. To meet these challenges, future research should focus more on the following aspects, from agricultural production to processing and comprehensive utilization, aiming to optimize the entire carrot value chain and promote sustainable and innovative practices within the industry.

The agricultural research focus encompasses a range of areas including breeding and genetics, agronomy and crop management, pest and disease management, and climate adaptation. This involves developing new carrot varieties with improved traits, studying optimal cultivation practices, researching integrated pest management strategies, and investigating climate‐resilient cultivation techniques. The innovation of carrot germplasm resources is of utmost importance. The use of raw materials is crucial for the processing of carrots. Currently, the market relies predominantly on fresh varieties. Urgent research and development are needed for carrot varieties suitable for processing, particularly for MP carrot production, juice extraction, and plant‐derived nutrient extraction. Furthermore, the processing of carrots encompasses food preservation and processing technologies, the development of value‐added products, and ensuring the quality and safety of processed carrot products. There is an urgent need to advance carrot processing technology because the biochemical changes during processing impact the appearance, texture, nutrition, and safety of carrot products. Hence, there is an essential need to accelerate the development of technologies that effectively control detrimental reactions, preserve nutrients, and increase the quality of processed carrots. The focus should also include reducing processing costs and enhancing global applicability. Lastly, given the expanding fresh market, addressing transportation conditions and optimizing cold chain equipment for carrot products are critically needed. Finally, the comprehensive utilization aspect involves the investigation of waste utilization, circular economy approaches, and economic and environmental impact assessments, aiming to create sustainable, circular economy models for carrot production, processing, and utilization. This includes exploring innovative techniques for preserving carrot quality during processing, developing new carrot‐based products, and evaluating the economic and environmental implications of comprehensive carrot utilization methods.

## Author Contributions

**Haiyan Ding:** conceptualization; data curation; formal analysis; investigation; methodology; funding acquisition; **Menglong Liu:** conceptualization; data curation; formal analysis; investigation; project administration.

## Conflicts of Interest

The authors declare no conflicts of interest.

## Data Availability

The data that support the findings of this study are available from the corresponding author upon reasonable request.

## References

[fsn34542-bib-0001] Ahmad, T. , M. Cawood , Q. Iqbal , et al. 2019. “Phytochemicals in *Daucus carota* and Their Health Benefits‐Review Article.” Foods 8, no. 9: 424–446. 10.3390/foods8090424. 31546950 PMC6770766

[fsn34542-bib-0002] Aimaretti, N. R. , C. V. Ybalo , M. L. Rojas , F. J. Plou , and J. C. Yori . 2012. “Production of Bioethanol From Carrot Discards.” Bioresource Technology 123: 727–732. 10.1016/j.biortech.2012.08.035. 22975251

[fsn34542-bib-0003] Akter, J. , J. Hassan , M. M. Rahman , M. S. Biswas , H. I. Khan , and M. M. Rahman Rajib . 2024. “Colour, Nutritional Composition and Antioxidant Properties of Dehydrated Carrot (*Daucus carota Var. sativus*) Using Solar Drying Techniques and Pretreatments.” Heliyon 10: e24165. 10.1016/j.heliyon.2024.e24165. 38293496 PMC10825429

[fsn34542-bib-0004] Alam, M. R. , J. G. Lyng , D. Frontuto , F. Marra , and L. Cinquanta . 2018. “Effect of Pulsed Electric Field Pretreatment on Drying Kinetics, Colour, and Texture of Parsnip and Carrot.” Journal of Food Science 83, no. 8: 2159–2166. 10.1111/1750-3841.14216. 30035307

[fsn34542-bib-0005] Aliyev, S. , M. Khalilov , R. Saidov , G. Mammadov , and G. Allahverdiyeva . 2021. “Comparative Assessment of the Effect of the Degree of Grinding of Vegetables (Carrots) on the Yield of Juices and Puree.” Eastern‐European Journal of Enterprise Technologies 6, no. 11: 60–67. 10.15587/1729-4061.2021.247669.

[fsn34542-bib-0006] Almatroodi, S. A. , A. Almatroudi , H. O. A. Alharbi , A. A. Khan , and A. H. Rahmani . 2024. “Effects and Mechanisms of Luteolin, a Plant‐Based Flavonoid, in the Prevention of Cancers via Modulation of Inflammation and Cell Signalling Molecules.” Molecules 29: 1093. 10.3390/molecules29051093. 38474604 PMC10934766

[fsn34542-bib-0007] Alvarado‐Ramírez, M. , J. Santana‐Gálvez , A. Santacruz , et al. 2018. “Using a Functional Carrot Powder Ingredient to Produce Sausages With High Levels of Nutraceuticals.” Journal of Food Science 83, no. 9: 2351–2361. 10.1111/1750-3841.14319. 30101977

[fsn34542-bib-0008] Aniceto, J. P. S. , V. H. Rodrigues , I. Portugal , and C. M. Silva . 2022. “Valorization of Tomato Residues by Supercritical Fluid Extraction.” PRO 10, no. 1: 28. 10.3390/pr10010028.

[fsn34542-bib-0009] Ariahu, C. C. , O. S. Kamaldeen , and M. I. Yusufu . 2021. “Kinetic and Thermodynamic Studies on the Degradation of Carotene in Carrot Powder Beads.” Journal of Food Engineering 288: 110145. 10.1016/j.jfoodeng.2020.110145.

[fsn34542-bib-0010] Attokaran, M. 2017. Natural Food Flavors and Colourants: Carrot, 2nd ed. Hoboken, NJ: John Wiley & Sons Inc. 10.1002/9781119114796.

[fsn34542-bib-0011] Balkrishna, A. , M. Joshi , S. Gupta , et al. 2024. “Dissecting the Natural Phytochemical Diversity of Carrot Roots With Its Colour Using High Performance Liquid Chromatography and UV‐Visible Spectrophotometry.” Heliyon 10, no. 16: e35918. 10.1016/j.heliyon.2024.e35918. 39220899 PMC11365394

[fsn34542-bib-0012] Bansal, V. , K. Jabeen , P. S. Rao , P. Prasad , and S. K. Yadav . 2019. “Effect of High Pressure Processing (HPP) on Microbial Safety, Physicochemical Properties, and Bioactive Compounds of Whey‐Based Sweet Lime (Whey‐Lime) Beverage.” Journal of Food Measurement and Characterization 13, no. 1: 454–465. 10.1007/s11694-018-9959-1.

[fsn34542-bib-0013] Bas‐Bellver, C. , C. Barrera , N. Betoret , and L. Seguí . 2023. “Effect of Processing and In Vitro Digestion on Bioactive Constituents of Powdered IV Range Carrot (*Daucus carota* L.) Wastes.” Food 12, no. 4: 731. 10.3390/foods12040731. PMC9955751 36832803

[fsn34542-bib-0014] Bhandari, S. , J. Rhee , C. Choi , et al. 2022. “Morphological and Biochemical Variation in Carrot Genetic Resources Grown Under Open Field Conditions: The Selection of Functional Genotypes for a Breeding Program.” Agronomy 12, no. 3: 553–572. 10.3390/agronomy12030553.

[fsn34542-bib-0015] Bhandari, S. R. , C. S. Choi , J. Rhee , et al. 2022. “Influence of Root Colour and Tissue on Phytochemical Contents and Antioxidant Activities in Carrot Genotypes.” Food 12, no. 1: 120. 10.3390/foods12010120. PMC9818746 36613336

[fsn34542-bib-0016] Bhargava, N. , V. S. Sharanagat , R. S. Mor , and K. Kumar . 2020. “Active and Intelligent Biodegradable Packaging Films Using Food and Food Waste‐Derived Bioactive Compounds: A Review.” Trends in Food Science & Technology 105: 385–401. 10.1016/j.tifs.2020.09.015.

[fsn34542-bib-0017] Bhatt, T. , and K. Patel . 2020. “Carotenoids: Potent to Prevent Diseases Review.” Natural Products and Bioprospecting 10, no. 3: 109–117. 10.1007/s13659-020-00244-2. 32405969 PMC7253555

[fsn34542-bib-0018] Cankurt, H. , M. Cavus , and O. Guner . 2024. “The Use of Carrot Fiber and Some Gums in the Production of Block‐Type Melting Cheese.” Food 13, no. 13: 2035. 10.3390/foods13132035. PMC11241037 38998541

[fsn34542-bib-0019] Caruso, G. , and S. Parisi . 2016. Foods of Non‐animal Origin: Chemistry and Technology of Ready‐To‐Eat Vegetable Foods. Cham, Switzerland: Springer. 10.1007/978-3-319-25649-8_3.

[fsn34542-bib-0020] Catania, P. , A. Comparetti , C. De Pasquale , G. Morello , and M. Vallone . 2020. “Effects of the Extraction Technology on Pomegranate Juice Quality.” Agronomy 10, no. 10: 1483. 10.3390/agronomy10101483.

[fsn34542-bib-0021] Chen, Q. , B. Wu , D. Pan , L. Sang , and B. Chang . 2021. “Beta‐Carotene and Its Protective Effect on Gastric Cancer.” World Journal of Clinical Cases 9, no. 23: 6591–6607. 10.12998/wjcc.v9.i23.6591. 34447808 PMC8362528

[fsn34542-bib-0022] Chizzola, R. 2010. “Composition of the Essential Oil From *Daucus carota* ssp. Carota Growing Wild in Vienna.” Journal of Essential Oil‐Bearing Plants 13, no. 1: 12–19. 10.1080/0972060X.2010.10643785.

[fsn34542-bib-0023] Choe, U. , Y. Li , B. Gao , et al. 2018. “Chemical Compositions of Cold‐Pressed Broccoli, Carrot, and Cucumber Seed Flours and Their In Vitro Gut Microbiota Modulatory, Anti‐Inflammatory, and Free Radical Scavenging Properties.” Journal of Agricultural and Food Chemistry 66, no. 35: 1–34. 10.1021/acs.jafc.8b03343. 30068076

[fsn34542-bib-0024] Chupawa, P. , W. Suksamran , D. Jaisut , F. Ronsse , and W. Duangkhamchan . 2022. “Combined Heat and Mass Transfer Associated With Kinetics Models for Analyzing Convective Stepwise Drying of Carrot Cubes.” Food 11, no. 24: 4045–4064. 10.3390/foods11244045. PMC9778106 36553787

[fsn34542-bib-0025] Clementz, A. , P. A. Torresi , J. S. Molli , D. Cardell , E. Mammarella , and J. C. Yori . 2019. “Novel Method for Valorization of By‐Products From Carrot Discards.” LWT 100: 374–380. 10.1016/j.lwt.2018.10.085.

[fsn34542-bib-0026] Condurso, C. , F. Cincotta , G. Tripodi , M. Merlino , F. Giarratana , and A. Verzera . 2020. “A New Approach for the Shelf‐Life Definition of Minimally Processed Carrots.” Postharvest Biology and Technology 163: 111138. 10.1016/j.postharvbio.2020.111138.

[fsn34542-bib-0027] De Andrade Lima, M. , D. Charalampopoulos , and A. Chatzifragkou . 2018. “Optimisation and Modelling of Supercritical CO_2_ Extraction Process of Carotenoids From Carrot Peels.” Journal of Supercritical Fluids 133: 94–102. 10.1016/j.supflu.2017.09.028.

[fsn34542-bib-0028] De Andrade Lima, M. , I. Kestekoglou , D. Charalampopoulos , and A. Chatzifragkou . 2019. “Supercritical Fluid Extraction of Carotenoids From Vegetable Waste Matrices.” Molecules 24, no. 3: 466. 10.3390/molecules24030466. 30696092 PMC6384789

[fsn34542-bib-0029] Debbarma, T. , S. Thangalakshmi , M. Tadakod , R. Singh , and A. Singh . 2021. “Comparative Analysis of Ohmic and Conventional Heat‐Treated Carrot Juice.” Journal of Food Processing and Preservation 45, no. 9: E15687. 10.1111/jfpp.15687.

[fsn34542-bib-0030] Demiray, E. , S. E. Karatay , S. Dönmez , and G. Dönmez . 2016. “The Usage of Carrot Pomace for Bioethanol Production.” Journal of the Chilean Chemical Society 61, no. 2: 2996–2998. 10.4067/S0717-97072016000200029.

[fsn34542-bib-0031] Dias, J. S. 2014. “Nutritional and Health Benefits of Carrots and Their Seed Extracts.” Food and Nutrition Sciences 5, no. 22: 2147–2156. 10.4236/fns.2014.522227.

[fsn34542-bib-0032] Dong, R. , W. Liao , J. Xie , et al. 2022. “Enrichment of Yogurt With Carrot Soluble Dietary Fiber Prepared by Three Physical Modified Treatments: Microstructure, Rheology and Storage Stability.” Innovative Food Science & Emerging Technologies 75: 102901. 10.1016/j.ifset.2021.102901.

[fsn34542-bib-0033] Ethaib, S. , R. Omar , S. M. M. Kamal , D. R. A. Biak , and S. L. Zubaidi . 2020. “Microwave‐Assisted Pyrolysis of Biomass Waste: A Mini Review.” PRO 8, no. 9: 1190. 10.3390/pr8091190.

[fsn34542-bib-0034] Feng, Y. , C. P. Tan , C. Zhou , et al. 2020. “Effect of Freeze‐Thaw Cycles Pretreatment on the Vacuum Freeze‐Drying Process and Physicochemical Properties of the Dried Garlic Slices.” Food Chemistry 324: 126883. 10.1016/j.foodchem.2020.126883. 32344350

[fsn34542-bib-0035] Finger, J. , I. Santos , G. Silva , M. Bernardino , U. Pinto , and D. Maffei . 2023. “Minimally Processed Vegetables in Brazil: An Overview of Marketing, Processing, and Microbiological Aspects.” Food 12, no. 11: 2259–2276. 10.3390/foods12112259. PMC10252532 37297503

[fsn34542-bib-0036] Forwood, D. L. , D. J. Innes , M. C. Parra , et al. 2023. “Feeding an Unsalable Carrot Total‐Mixed Ration Altered Bacterial Amino Acid Degradation in the Rumen of Lambs.” Scientific Reports 13, no. 1: 6942. 10.1038/s41598-023-34181-0. 37117259 PMC10147942

[fsn34542-bib-0037] Gajewski, M. , P. Szymczak , K. Elkner , A. Dąbrowska , A. Kret , and H. Danilčenko . 2007. “Some Aspects of Nutritive and Biological Value of Carrot Cultivars With Orange, Yellow and Purple‐Coloured Roots.” Vegetable Crops Research Bulletin 67: 149–161. 10.2478/v10032-007-0039-z.

[fsn34542-bib-0038] Gastélum Estrada, A. , A. Hurtado Romero , A. Santacruz , L. Cisneros Zevallos , and D. A. Jacobo Velázquez . 2020. “Sanitizing After Fresh‐Cutting Carrots Reduces the Wound‐Induced Accumulation of Phenolic Antioxidants Compared to Sanitizing Before Fresh‐Cutting.” Journal of the Science of Food and Agriculture 100, no. 13: 4995–4998. 10.1002/jsfa.10555. 32478414

[fsn34542-bib-0039] Gastélum‐Estrada, A. , G. Rabadán‐Chávez , E. Reza‐Zaldívar , et al. 2023. “Biofortified Beverage With Chlorogenic Acid From Stressed Carrots: Anti‐Obesogenic, Antioxidant, and Anti‐Inflammatory Properties.” Food 12, no. 21: 3959–3979. 10.3390/foods12213959. PMC10648175 37959079

[fsn34542-bib-0040] Gomaa, M. M. , M. A. Gomaa , and W. M. M. A. El‐All . 2021. “Quality Characteristics of Carrot (*Daucus carota* L.) Puree Supplemented With Quinoa (*Chenopodium quinoa* Willd.).” Asian Food Science Journal 20: 64–80. 10.9734/afsj/2021/v20i430288.

[fsn34542-bib-0041] González‐Peña, M. , A. Ortega‐Regules , C. Parrodi , and J. Lozada‐Ramírez . 2023. “Chemistry, Occurrence, Properties, Applications, and Encapsulation of Carotenoids‐a Review.” Plants 12, no. 2: 313–335. 10.3390/plants12020313. 36679026 PMC9865331

[fsn34542-bib-0043] He, J. J. , C. H. Chiu , M. Gavahian , C. T. Ho , and Y. L. Chu . 2022. “Development and Application of Edible Coating on Dried Pineapple Exposed to Electrical Blanching.” Journal of Food Processing and Preservation 46, no. 8: e16760. 10.1111/jfpp.16760.

[fsn34542-bib-0044] Hernández‐Acosta, E. , C. Muro , A. Y. Guadarrama‐Lezama , E. Gutierrez‐Cortez , and E. López‐Solórzano . 2024. “Valorization of Black Carrot Industrial Residues for the Anthocyanin Pigment Production.” Waste and Biomass Valorization 15: 4071–4086. 10.1007/s12649-024-02424-4.

[fsn34542-bib-0045] Hernández‐Hernández, H. M. , L. Moreno‐Vilet , and S. J. Villanueva‐Rodríguez . 2019. “Current Status of Emerging Food Processing Technologies in Latin America: Novel Non‐Thermal Processing.” Innovative Food Science & Emerging Technologies 58: 102233. 10.1016/j.ifset.2019.102233.

[fsn34542-bib-0046] Howarth, M. S. , J. R. Brandon , S. W. Searcy , and N. Kehtarnavaz . 2015. “Estimation of Tip Shape for Carrot Classification by Machine Vision.” Journal of Agricultural Engineering Research 53, no. 2: 123–139. 10.1016/0021-8634(92)80078-7.

[fsn34542-bib-0047] Hwang, C. C. , H. I. Chien , Y. C. Lee , et al. 2023. “Effect of High‐Pressure Processing on the Qualities of Carrot Juice During Cold Storage.” Food 12, no. 16: 3107. 10.3390/foods12163107. PMC10453467 37628106

[fsn34542-bib-0048] Ignaczak, A. , A. Salamon , J. Kowalska , A. Marzec , and H. Kowalska . 2023. “Influence of Pre‐Treatment and Drying Methods on the Quality of Dried Carrot Properties as Snacks.” Molecules 28, no. 17: 6407–6429. 10.3390/molecules28176407. 37687236 PMC10490186

[fsn34542-bib-0049] Ikram, A. , A. Rasheed , A. Ahmad Khan , et al. 2024. “Exploring the Health Benefits and Utility of Carrots and Carrot Pomace: A Systematic Review.” International Journal of Food Properties 27, no. 1: 180–193. 10.1080/10942912.2023.2301569.

[fsn34542-bib-0050] Ismail, J. , W. Shebaby , J. Daher , et al. 2023. “The Wild Carrot (*Daucus carota*): A Phytochemical and Pharmacological Review.” Plants 13, no. 1: 93–117. 10.3390/plants13010093. 38202401 PMC10781147

[fsn34542-bib-0051] Jeż, M. , W. Błaszczak , D. Zielińska , W. Wiczkowski , and I. Białobrzewski . 2019. “Carotenoids and Lipophilic Antioxidant Capacities of Tomato purées as Affected by High Hydrostatic Pressure Processing.” International Journal of Food Science & Technology 55, no. 1: 65–73. 10.1111/ijfs.14231.

[fsn34542-bib-0052] Johra, F. T. , A. K. Bepari , A. T. Bristy , and H. M. Reza . 2020. “A Mechanistic Review of Beta‐Carotene, Lutein, and Zeaxanthin in Eye Health and Disease.” Antioxidants 9, no. 11: 1046. 10.3390/antiox9111046. 33114699 PMC7692753

[fsn34542-bib-0053] Kasale, K. , U. Malagi , and K. R. Naik . 2018. “Physico‐Chemical Composition and Acceptability of Newer Carrot Germplasms.” International Journal of Current Microbiology and Applied Sciences 7, no. 12: 705–715. 10.20546/IJCMAS.2018.712.087.

[fsn34542-bib-0054] Kaur, P. , J. Subramanian , and A. Singh . 2022. “Green Extraction of Bioactive Components From Carrot Industry Waste and Evaluation of Spent Residue as an Energy Source.” Scientific Reports 12, no. 1: 16607. 10.1038/s41598-022-20971-5. 36198728 PMC9534898

[fsn34542-bib-0055] Kee, S. H. , J. B. V. Chiongson , J. P. Saludes , S. Vigneswari , S. Ramakrishna , and K. Bhubalan . 2021. “Bioconversion of Agro‐Industry Sourced Biowaste Into Biomaterials via Microbial Factories—A Viable Domain of Circular Economy.” Environmental Pollution 271: 116311. 10.1016/j.envpol.2020.116311. 33383425

[fsn34542-bib-0056] Kiros, E. , E. Seifu , G. Bultosa , and W. K. Solomon . 2016. “Effect of Carrot Juice and Stabilizer on the Physicochemical and Microbiological Properties of Yoghurt.” LWT 69: 191–196. 10.1016/j.lwt.2016.01.026.

[fsn34542-bib-0057] Kumar, A. , P. Choudhary , S. Pandhi , D. C. Rai , and V. Paul . 2020. “Process Optimization for the Production of Ready‐To‐Cook Carrot Halwa.” Indian Journal of Dairy Science 73, no. 4: 285–291. 10.33785/ijds.2020.v73i04.001.

[fsn34542-bib-0058] Lee, M. , N. H. Jeong , and B. S. Jang . 2015. “Antioxidative Activity and Antiaging Effect of Carrot Glycoprotein.” Journal of Industrial and Engineering Chemistry (Seoul, Korea) 25: 216–221. 10.1016/j.jiec.2014.10.037.

[fsn34542-bib-0059] Lee, M. J. , B. S. Jang , and N. H. Jeong . 2013. “Physicochemical Characteristics of Carrot Glycoprotein.” Journal of the Korean Industrial and Engineering Chemistry 24, no. 1: 62–66.

[fsn34542-bib-0060] Lepaus, B. M. , S. A. K. P. de Oliveira , A. F. Spaviero , P. S. Daud , and J. F. B. de São José . 2023. “Thermosonication of Orange‐Carrot Juice Blend: Overall Quality During Refrigerated Storage, and Sensory Acceptance.” Molecules 28, no. 5: 2196. 10.3390/molecules28052196. 36903442 PMC10005015

[fsn34542-bib-0061] Li, G. , Q. Wang , and H. Zhou . 2023. “Research on the Application of Vacuum Freeze‐Drying Technology for Food.” E3S Web of Conferences 370: 1004. 10.1051/e3sconf/202337001004.

[fsn34542-bib-0062] Li, L. , X. Chen , W. Cao , et al. 2024. “Effects of Freeze Thaw Pre‐Treatment With Different Freezing Methods on the Microwave Freeze Drying of Carrots.” International Journal of Food Science and Technology 59: 7181–7192. 10.1111/ijfs.17439.

[fsn34542-bib-0063] Li, M. N. , Q. B. Yao , H. R. Zhang , et al. 2024. “Impact of Different Lactic Acid Bacteria on Nitrite Degradation and Quality of Fermented Carrot.” International Journal of Food Science and Technology 59: 6501–6512. 10.1111/ijfs.17397.

[fsn34542-bib-0064] Ling, J. G. , X. T. Xuan , N. Yu , et al. 2020. “High Pressure‐Assisted Vacuum‐Freeze Drying: A Novel, Efficient Way to Accelerate Moisture Migration in Shrimp Processing.” Journal of Food Science 85, no. 4: 1167–1176. 10.1111/1750-3841.15027. 32275070

[fsn34542-bib-0065] Liu, J. , J. Bi , X. Liu , et al. 2023. “Polygalacturonase Treatment Affects Carotenoid Absorption From Veggie Juice.” Food Chemistry 415: 135748. 10.1016/j.foodchem.2023.135748. 36854238

[fsn34542-bib-0066] López‐Gámez, G. , P. Elez‐Martínez , O. Martín‐Belloso , and R. Soliva‐Fortuny . 2020. “Pulsed Electric Fields Affect Endogenous Enzyme Activities, Respiration and Biosynthesis of Phenolic Compounds in Carrots.” Postharvest Biology and Technology 168: 111284. 10.1016/j.postharvbio.2020.111284.

[fsn34542-bib-0067] Lu, X. , H. Hou , D. Fang , Q. Hu , J. Chen , and L. Zhao . 2021. “Identification and Characterization of Volatile Compounds in *Lentinula edodes* During Vacuum Freeze‐Drying.” Journal of Food Biochemistry 0: e13814. 10.1111/jfbc.13814. 34089191

[fsn34542-bib-0068] Luzardo‐Ocampo, I. , A. K. Ramírez‐Jiménez , J. Yañez , L. Mojica , and D. A. Luna‐Vital . 2021. “Technological Applications of Natural Colourants in Food Systems: A Review.” Food 10, no. 3: 634. 10.3390/foods10030634. PMC8002548 33802794

[fsn34542-bib-0069] Ma, J. , C. Chen , J. Ma , W. Ma , and J. Yang . 2020. “Analysis of Bioactive Compounds and Antioxidant Capacities in Different Varieties of Carrots.” Journal of Physics Conference Series 1549, no. 3: 32054. 10.1088/1742-6596/1549/3/032054.

[fsn34542-bib-0070] Ma, T. , J. Luo , C. Tian , et al. 2015. “Influence of Technical Processing Units on Chemical Composition and Antimicrobial Activity of Carrot (*Daucus carrot* L.) Juice Essential Oil.” Food Chemistry 170: 394–400. 10.1016/j.foodchem.2014.08.018. 25306362

[fsn34542-bib-0071] Makki, K. , E. C. Deehan , J. Walter , and F. Bäckhed . 2018. “The Impact of Dietary Fiber on Gut Microbiota in Host Health and Disease.” Cell Host & Microbe 23, no. 6: 705–715. 10.1016/j.chom.2018.05.012. 29902436

[fsn34542-bib-0072] Marcelino, G. , D. J. Machate , K. D. C. Freitas , et al. 2020. “β‐Carotene: Preventive Role for Type 2 Diabetes Mellitus and Obesity: A Review.” Molecules 25, no. 24: 5803. 10.3390/molecules25245803. 33316948 PMC7763535

[fsn34542-bib-0073] Megali, H. K. H. , S. M. I. Darwish , H. I. Abedel‐Hakim , and M. Abdelrahman . 2020. “Utilization of Red Carrot Roots (*Daucus carota* L.) by‐Products as a Source of Natural Pigments.” Assiut Journal of Agricultural Sciences 1, no. 50: 50–61. 10.21608/ajas.2019.33458.

[fsn34542-bib-0074] Miękus, N. , A. Iqbal , K. Marszałek , C. Puchalski , and A. Świergiel . 2019. “Green Chemistry Extractions of Carotenoids From *Daucus carota* L.‐Supercritical Carbon Dioxide and Enzyme‐Assisted Methods.” Molecules 24, no. 23: 4339. 10.3390/molecules24234339. 31783600 PMC6930531

[fsn34542-bib-0075] Mohammedi, H. , S. Mecherara‐Idjeri , Y. Foudil‐Cherif , and A. Hassani . 2015. “Chemical Composition and Antioxidant Activity of Essential Oils From Algerian *Daucus carota* L. subsp. *carota* Aerial Parts.” Journal of Essential Oil Bearing Plants 18, no. 4: 873–883. 10.1080/0972060X.2015.1010596.

[fsn34542-bib-0076] Moradi, R. , M. Kashefi‐Alasl , R. Marandi , E. Salahi , and S. Moradidehaqi . 2021. “Application of Box‐Behnken Design and Response Surface Methodology of Acid Red 18 Adsorption Onto PAC (Synthesized Carrot Waste) Coated With Fe_3_O_4_ Nanoparticles From Aquatic Solution: Kinetic and Isotherm Studies.” Archives of Hygiene Sciences 10: 30–48. 10.52547/archhygsci.10.1.30.

[fsn34542-bib-0077] Munteanu‐Ichim, R. A. , C. M. Canja , M. Lupu , C. L. Bădărău , and F. Matei . 2024. “Tradition and Innovation in Yoghurt From a Functional Perspective—A Review.” Fermentation 10: 357. 10.3390/fermentation10070357.

[fsn34542-bib-0078] Muwakhid, B. , U. Kalsum , and A. M. Wati . 2021. “Effect of Different Feed Additives on Ensiled Carrot Straw as an Animal Feed.” Jurnal Peternakan Indonesia 23, no. 3: 240–246. 10.25077/jpi.23.3.240-246.2021.

[fsn34542-bib-0079] Nahar, L. , W. Alsheikh , K. J. Ritchie , and S. D. Sarker . 2024. “Naturally Occurring Eugenin: Biosynthesis, Distribution, Bioactivity, and Therapeutic Potential.” Phytochemistry Letters 61: 191–197. 10.1016/j.phytol.2024.04.017.

[fsn34542-bib-0080] Naik, A. S. , D. Suryawanshi , M. Kumar , and R. Waghmare . 2021. “Ultrasonic Treatment: A Cohort Review on Bioactive Compounds, Allergens and Physico‐Chemical Properties of Food.” Current Research in Food Science 4: 470–477. 10.1016/j.crfs.2021.07.003. 34286286 PMC8280479

[fsn34542-bib-0081] Negri Rodríguez, L. M. , R. Arias , T. Soteras , et al. 2021. “Comparison of the Quality Attributes of Carrot Juice Pasteurized by Ohmic Heating and Conventional Heat Treatment.” LWT 145: 111255. 10.1016/j.lwt.2021.111255.

[fsn34542-bib-0082] Nguyn, H. V. H. 2015. Handbook of Vegetable Preservation and Processing: Carrot Processing, 2nd ed. Florida, United States: CRC Press, Taylor & Francis Group.

[fsn34542-bib-0083] Özcan, T. , and E. Yıldız . 2016. “Determination of Textural and Sensory Properties of Yogurt Produced With the Vegetable Puree.” Turkish Journal of Agriculture: Food Science and Technology 4, no. 7: 579–587. 10.24925/turjaf.v4i7.579-587.719.

[fsn34542-bib-0084] Palacios‐Velásquez, A. , V. Quispe‐Coquil , E. M. Casimiro‐Soriano , K. M. Tapia‐Zarate , and A. R. Huamán‐De La Cruz . 2023. “Acquisition, Characterization, and Optimization of Distilled Bioethanol Generated From Fermented Carrot (*Daucus carota*) Residues.” Fermentation 9, no. 10: 867. 10.3390/fermentation9100867.

[fsn34542-bib-0085] Pandey, P. , K. Grover , T. S. Dhillon , N. Chawla , and A. Kaur . 2024. “Development and Quality Evaluation of Polyphenols Enriched Black Carrot (*Daucus carota* L.) Powder Incorporated Bread.” Heliyon 10: e25109. 10.1016/j.heliyon.2024.e25109. 38322869 PMC10844063

[fsn34542-bib-0086] Pandey, P. , K. Grover , T. S. Dhillon , A. Kaur , and M. Javed . 2021. “Evaluation of Polyphenols Enriched Dairy Products Developed by Incorporating Black Carrot (*Daucus carota* L.) Concentrate.” Heliyon 7, no. 5: e6880. 10.1016/j.heliyon.2021.e06880. PMC8113845 34013075

[fsn34542-bib-0087] Paparella, A. , P. R. Kongala , A. Serio , et al. 2024. “Challenges and Opportunities in the Sustainable Improvement of Carrot Production.” Plants 13: 2092. 10.3390/plants13152092. 39124210 PMC11314595

[fsn34542-bib-0088] Piscopo, A. , A. Zappia , M. P. Princi , et al. 2019. “Quality of Shredded Carrots Minimally Processed by Different Dipping Solutions.” Journal of Food Science and Technology 56, no. 5: 2584–2593. 10.1007/s13197-019-03741-6. 31168140 PMC6525718

[fsn34542-bib-0089] Porto Dalla Costa, A. , R. Cruz Silveira Thys , A. De Oliveira Rios , and S. Hickmann Flôres . 2016. “Carrot Flour From Minimally Processed Residue as Substitute of β‐Carotene Commercial in Dry Pasta Prepared With Common Wheat (*Triticum aestivum*).” Journal of Food Quality 39, no. 6: 590–598. 10.1111/jfq.12253.

[fsn34542-bib-0090] Prerana, S. , and D. Anupama . 2020. “Influence of Carrot Puree Incorporation on Quality Characteristics of Instant Noodles.” Journal of Food Process Engineering 43, no. 3: e13270. 10.1111/jfpe.13270.

[fsn34542-bib-0091] Priyanka, P. , and S. Khanam . 2019. “Supercritical CO_2_ Extraction of Carrot Seed Oil: Screening, Optimization and Economic Analysis.” International Journal of Environmental Science and Technology 17, no. 4: 2311–2324. 10.1007/s13762-019-02497-y.

[fsn34542-bib-0092] Purkiewicz, A. , J. Ciborska , M. Tańska , et al. 2020. “The Impact of the Method Extraction and Different Carrot Variety on the Carotenoid Profile, Total Phenolic Content and Antioxidant Properties of Juices.” Plants 9, no. 12: 1759. 10.3390/plants9121759. 33322599 PMC7764007

[fsn34542-bib-0093] Qin, Y. H. , Y. Y. Hang , R. Y. Feng , and Y. C. Qin . 2018. “Processing Technology of Composite Vegetable Paper for Carrot‐Spinach.” Farm Products Processing 17: 10–12. 10.16693/j.cnki.1671-9646(X).2018.09.004.

[fsn34542-bib-0094] Que, F. , X. Hou , G. Wang , et al. 2019. “Advances in Research on the Carrot, an Important Root Vegetable in the *Apiaceae* Family.” Horticulture Research 6, no. 1: 69. 10.1038/s41438-019-0150-6. 31231527 PMC6544626

[fsn34542-bib-0095] Ratajczak, K. , J. Staninska‐Pięta , J. Czarny , P. Cyplik , Ł. Wolko , and A. Piotrowska‐Cyplik . 2022. “Effect of Processing Treatment and Modified Atmosphere Packing on Carrot's Microbial Community Structure by Illumina Miseq Sequencing.” Molecules 27, no. 9: 2830–2853. 10.3390/molecules27092830. 35566181 PMC9103152

[fsn34542-bib-0096] Ravula, S. R. , D. Arepally , G. Sandeep , S. R. Munagala , and P. R. Ravula . 2017. “Effect of Process Variables on Osmotic Dehydration of Carrot Slices.” International Journal of Chemical Studies 5, no. 4: 1280–1284.

[fsn34542-bib-0097] Ren, X. , M. S. Ghazani , H. Zhu , et al. 2022. “Challenges and Opportunities in Microwave‐Assisted Catalytic Pyrolysis of Biomass: A Review.” Applied Energy 315: 118970. 10.1016/j.apenergy.2022.118970.

[fsn34542-bib-0098] Rodríguez, G. , L. Sheila , and R. Vijaya . 2021. “Green Extraction Techniques From Fruit and Vegetable Waste to Obtain Bioactive Compounds—A Review.” Critical Reviews in Food Science and Nutrition 62: 6446–6466. 10.1080/10408398.2021.1901651. 33792417

[fsn34542-bib-0099] Roshana, M. R. , and T. Mahendran . 2019. “Nutritional and Sensory Evaluation of Carrot Flour‐Incorporated Complementary Food Mixtures for Infants.” Sri Lanka Journal of Food and Agriculture 5: 27–32. 10.4038/sljfa.v5i2.74.

[fsn34542-bib-0100] Sadeghi, S. , R. Bahrami , F. Raisi , Z. Rampisheh , A. Ghobadi , and E. Akhtari . 2020. “Evaluation of the Effect of Carrot Seed (*Daucus carota*) in Women of Fertile Age With Hypoactive Sexual Desire Disorder: A Randomized Double‐Blind Clinical Trial.” Complementary Therapies in Medicine 54: 102543. 10.1016/j.ctim.2020.102543. 33183662

[fsn34542-bib-0101] Sagar, V. R. 2015. “Effect of Drying Methods and Storage on Quality of Ready‐To‐Eat Dehydrated Carrot Shreds.” Journal of Agricultural Engineering 52, no. 1: 26–30. 10.52151/jae2015521.1568.

[fsn34542-bib-0102] Salehi, F. , M. Kashaninejad , E. Akbari , S. M. Sobhani , and F. Asadi . 2015. “Potential of Sponge Cake Making Using Infrared–Hot Air Dried Carrot.” Journal of Texture Studies 47, no. 1: 34–39. Portico. 10.1111/jtxs.12165.

[fsn34542-bib-0104] Say, D. , İ. B. Saydam , and N. Güzeler . 2022. “Effects of Freeze‐Dried Black Carrot Fiber Addition on the Physicochemical, Colour, Sensory Attributes, and Mineral Contents of Ayran.” Journal of Food Processing and Preservation 46, no. 12: e17225. 10.1111/jfpp.17225.

[fsn34542-bib-0105] Šeregelj, V. , L. Pezo , O. Šovljanski , et al. 2021. “New Concept of Fortified Yogurt Formulation With Encapsulated Carrot Waste Extract.” LWT 138: 110732. 10.1016/j.lwt.2020.110732.

[fsn34542-bib-0106] Shang, Y. Y. , G. J. Tian , Z. Y. Huang , and W. Chen . 2012. “Research of the Processing Technology of Carrot Paper.” Food Research and Development 33, no. 2: 68–71.

[fsn34542-bib-0107] Sharma, K. D. , S. Karki , N. S. Thakur , and S. Attri . 2012. “Chemical Composition, Functional Properties and Processing of Carrot—A Review.” Journal of Food Science and Technology 49, no. 1: 22–32. 10.1007/s13197-011-0310-7. 23572822 PMC3550877

[fsn34542-bib-0108] Shirazi, S. L. , A. Koocheki , E. Milani , and M. Mohebbi . 2020. “Production of High Fiber Ready‐To‐Eat Expanded Snack From Barley Flour and Carrot Pomace Using Extrusion Cooking Technology.” Journal of Food Science and Technology 57, no. 6: 2169–2181. 10.1007/s13197-020-04252-5. 32431343 PMC7230109

[fsn34542-bib-0109] Silva, D. R. P. F. F. , T. A. P. Rocha‐Santos , and A. C. Duarte . 2016. “Supercritical Fluid Extraction of Bioactive Compounds.” TrAC Trends in Analytical Chemistry 76: 40–51. 10.1016/j.trac.2015.11.013.

[fsn34542-bib-0110] Simon, P. W. 2019. “Beyond the Genome: Carrot Production Trends, Research Advances, and Future Crop Improvement.” Acta Horticulturae 1264, no. 1: 1. 10.17660/actahortic.2019.1264.1.

[fsn34542-bib-0111] Singh, A. , U. Bhardwaj , R. Kaur , and P. Vyas . 2023. “Chemical Composition, Microencapsulation and Comparative Antimicrobial Studies of Unencapsulated and Encapsulated Carrot Seed Essential Oil.” Journal of Essential Oil Bearing Plants 26, no. 1: 232–243. 10.1080/0972060X.2023.2182712.

[fsn34542-bib-0112] Singh, M. N. , R. Srivastava , and D. I. Yadav . 2021. “Study of Different Varieties of Carrot and Its Benefits for Human Health: A Review.” Journal of Pharmacognosy and Phytochemistry 10, no. 1: 1293–1299. 10.22271/phyto.2021.v10.i1r.13529.

[fsn34542-bib-0113] Singh, S. K. , and V. Gangwar . 2020. “Drying Behavior of Osmo‐Convective Drying of Carrot Slices and Quality Characteristics of Dehydrated Products.” International Journal of Current Microbiology and Applied Sciences 9, no. 10: 1424–1431. 10.20546/ijcmas.2020.910.169.

[fsn34542-bib-0114] Śmigielski, K. B. , M. Majewska , A. Kunicka‐Styczyñska , and R. Gruska . 2014a. “The Effect of Ultrasound‐Assisted Maceration on the Bioactivity, Chemical Composition and Yield of Essential Oil From Waste Carrot Seeds (*Daucus carota*).” Journal of Essential Oil Bearing Plants 17, no. 6: 1075–1086. 10.1080/0972060X.2014.931253.

[fsn34542-bib-0115] Śmigielski, K. B. , M. Majewska , A. Kunicka‐Styczyńska , M. Szczęsna‐Antczak , R. Gruska , and Ł. Stańczyk . 2014b. “Effect of Enzyme‐Assisted Maceration on Bioactivity, Quality and Yield of Essential Oil From Waste Carrot (*Daucus carota*) Seeds.” Journal of Food Quality 37, no. 4: 219–228. 10.1111/jfq.12092.

[fsn34542-bib-0116] Stanojević, J. , Z. S. Ilić , L. Stanojević , et al. 2023. “Essential Oil Yield, Composition, and Antioxidant Activity in Two Umbel Maturity Stages of Wild Carrot (*Daucus carota* L. ssp. Carota) From Montenegro.” Horticulturae 9, no. 3: 328. 10.3390/horticulturae9030328.

[fsn34542-bib-0117] Starek‐Wójcicka, A. , A. Sagan , P. Terebun , et al. 2023. “Quality of Tomato Juice as Influenced by Non‐Thermal Air Plasma Treatment.” Applied Sciences 13, no. 1: 578–591. 10.3390/app13010578.

[fsn34542-bib-0118] Stoica, F. , R. N. Rațu , I. Motrescu , et al. 2024. “Application of Pomace Powder of Black Carrot as a Natural Food Ingredient in Yoghurt.” Food 13, no. 7: 1130. 10.3390/foods13071130. PMC11011250 38611434

[fsn34542-bib-0120] Sun, T. , P. Simon , and S. Tanumihardjo . 2009. “Antioxidant Phytochemicals and Antioxidant Capacity of Biofortified Carrots (*Daucus carota* L.) of Various Colours.” Journal of Agricultural and Food Chemistry 57, no. 10: 4142–4147. 10.1021/jf9001044. 19358535

[fsn34542-bib-0121] Swarup, C. , R. Choudhury , M. Mostofa , et al. 2023. “Comparative Root Transcriptome Profiling and Gene Regulatory Network Analysis Between Eastern and Western Carrot (*Daucus carota* L.) Cultivars Reveals Candidate Genes for Vascular Tissue Patterning.” Plants 12, no. 19: 3449–3473. 10.3390/plants12193449. 37836190 PMC10575051

[fsn34542-bib-0122] Szczepańska, J. , C. A. Pinto , S. Skąpska , J. A. Saraiva , and K. Marszałek . 2021. “Effect of Static and Multi‐Pulsed High Pressure Processing on the Rheological Properties, Microbial and Physicochemical Quality, and Antioxidant Potential of Apple Juice During Refrigerated Storage.” LWT 150: 112038. 10.1016/j.lwt.2021.112038.

[fsn34542-bib-0123] Tabtiang, S. , P. Umroong , and S. Soponronnarit . 2022. “Comparative Study of the Effects of Thermal Blanching Pretreatments and Puffing Temperature Levels on the Microstructure and Qualities of Crisp Banana Slices.” Journal of Food Process Engineering 45, no. 1: e13931. 10.1111/jfpe.13931.

[fsn34542-bib-0124] Tanguler, H. , A. Cankaya , E. Agcam , and H. Uslu . 2021. “Effect of Temperature and Production Method on Some Quality Parameters of Fermented Carrot Juice (Shalgam).” Food Bioscience 41, no. 3: 100973. 10.1016/j.fbio.2021.100973.

[fsn34542-bib-0125] Tian, Z. , T. Dong , S. Wang , et al. 2024. “A Comprehensive Review on Botany, Chemical Composition and the Impacts of Heat Processing and Dehydration on the Aroma Formation of Fresh Carrot.” Food Chemistry: X 8, no. 22: 101201. 10.1016/j.fochx.2024.101201. PMC10972819 38550883

[fsn34542-bib-0126] Turturică, M. , E. Enachi , V. Barbu , and G. E. Bahrim . 2021. “Development of an Innovative Frozen Dairy Product Fortified With Carrot Extract.” Annals of the University Dunarea de Jos of Galati, Fascicle VI‐Food Technology 45, no. 2: 77–95. 10.35219/foodtechnology.2021.2.06.

[fsn34542-bib-0127] Utami, M. M. D. , A. C. Dewi , N. Ningsih , F. B. R. Maulana , and S. Islamianda . 2023. “Addition of Avocado (*Persea americana*) Leaf Extract and Carrot (*Daucus carota*) Leaf Extract in Starter Phase Broiler Feed for Production of Low‐Fat Meat for Elderly.” IOP Conference Series: Earth and Environmental Science 1168, no. 1: 12025. 10.1088/1755-1315/1168/1/012025.

[fsn34542-bib-0128] Valerga, L. , R. E. González , M. B. Pérez , A. Concellón , and P. F. Cavagnaro . 2023. “Differential and Cultivar‐Dependent Antioxidant Response of Whole and Fresh‐Cut Carrots of Different Root Colours to Postharvest Uv‐C Radiation.” Plants 12, no. 6: 1297. 10.3390/plants12061297. 36986985 PMC10053824

[fsn34542-bib-0129] Varshney, K. , and K. B. Mishra . 2022. “An Analysis of Health Benefits of Carrot.” International Journal of Innovative Research in Engineering and Management 9, no. 1: 211–214. 10.55524/ijirem.2022.9.1.40.

[fsn34542-bib-0130] Vénica, C. I. , M. J. Spotti , Y. L. Pavón , J. S. Molli , and M. C. Perotti . 2020. “Influence of Carrot Fibre Powder Addition on Rheological, Microstructure and Sensory Characteristics of Stirred‐Type Yogurt.” International Journal of Food Science & Technology 55, no. 5: 1916–1923. 10.1111/ijfs.14415.

[fsn34542-bib-0131] Wang, F. , G. Wang , X. Hou , M. Li , Z. Xu , and A. Xiong . 2018. “The Genome Sequence of ‘Kurodagosun’, a Major Carrot Variety in Japan and China, Reveals Insights Into Biological Research and Carrot Breeding.” Molecular Genetics and Genomics 293, no. 4: 861–871. 10.1007/s00438-018-1428-3. 29497811

[fsn34542-bib-0132] Wang, X. , D. Kong , Z. Ma , and R. Zhao . 2015. “Effect of Carrot Puree Edible Films on Quality Preservation of Fresh‐Cut Carrots.” Irish Journal of Agricultural and Food Research 54, no. 1: 64–71. 10.1515/ijafr-2015-0007.

[fsn34542-bib-0133] Wilczyński, K. , Z. Kobus , R. Nadulski , and M. Szmigielski . 2020. “Assessment of the Usefulness of the Twin‐Screw Press in Terms of the Pressing Efficiency and Antioxidant Properties of Apple Juice.” PRO 8, no. 1: 101. 10.3390/pr8010101.

[fsn34542-bib-0134] Xiao, N. , and Z. Y. Li . 2013. “Study of Production Process of Making Edible Vegetable Paper by Carrot.” Guangdong Agricultural Sciences 40, no. 2: 77–89. 10.16768/j.issn.1004-874x.2013.02.012.

[fsn34542-bib-0135] Ye, N. , P. Hu , S. Xu , et al. 2018. “Preparation and Characterization of Antioxidant Peptides From Carrot Seed Protein.” Journal of Food Quality 2018: 8579094. 10.1155/2018/8579094.

[fsn34542-bib-0136] Ying, D. , L. Sanguansri , L. Cheng , and M. A. Augustin . 2021. “Nutrient‐Dense Shelf‐Stable Vegetable Powders and Extruded Snacks Made From Carrots and Broccoli.” Food 10, no. 10: 2298. 10.3390/foods10102298. PMC8535146 34681346

[fsn34542-bib-0137] Ying, Z. , W. Ren , X. Fang , Q. Li , and W. Zhou . 2012. “Optimization Study on Adhesive of Carrot‐ Potato Compound Vegetable Paper by Response Surface Methodology.” Farm Products Processing 11: 43–46. 10.3969/j.issn.1671-9646(C).2012.11.001.

[fsn34542-bib-0138] Yu, J. X. , W. Z. Hu , M. R. Zhao , X. S. Sun , and K. X. Hao . 2019. “*Research Progress on the Application of Physical Treatment Methods in Fresh‐Cut Carrot Preservation*. The 16th Annual Meeting of China Food Science and Technology Association and the 10th Sino‐US Food Industry Forum, China.” https://kns.cnki.net/kcms/detail/detail.aspx?FileName=ZGSP201911001418&DbName=IPFD2019.

[fsn34542-bib-0139] Yusuf, E. , A. Wojdyło , J. Oszmiański , and P. Nowicka . 2021. “Nutritional, Phytochemical Characteristics and In Vitro Effect on α‐Amylase, α‐Glucosidase, Lipase, and Cholinesterase Activities of 12 Coloured Carrot Varieties.” Food 10, no. 4: 808. 10.3390/foods10040808. PMC8070462 33918549

[fsn34542-bib-0140] Zhang, Z. , Q. Wei , M. Nie , et al. 2018. “Microstructure and Bioaccessibility of Different Carotenoid Species as Affected by Hot Air Drying: Study on Carrot, Sweet Potato, Yellow Bell Pepper and Broccoli.” LWT 96: 357–363. 10.1016/j.lwt.2018.05.061.

[fsn34542-bib-0141] Zhuang, F. Y. , H. Hu , and Z. Fang . 2007. “The Concept Source and Market Development Prospect of Baby Carrot.” China Vegetable 03: 43–44.

[fsn34542-bib-0142] Zhuang, F. Y. , H. Hu , and Z. Fang . 2008. “Concept and Market Prospect of Micro Carrot.” Beijing Agriculture 17: 9–10.

